# Using high pressure to investigate the stability of a high entropy wurtzite structured (MnFeCuAgZnCd)S

**DOI:** 10.1038/s42004-025-01463-9

**Published:** 2025-03-05

**Authors:** Mark A. Buckingham, Joshua J. Shea, Kho Zhi Quan, Pok Man Ethan Lo, Joshua Swindell, Weichen Xiao, David J. Lewis, Alex S. Eggeman, Simon A. Hunt

**Affiliations:** Department of Materials, The University of Manchester, Oxford Road, M13 9PL Manchester, UK

**Keywords:** Materials chemistry, Phase transitions and critical phenomena, Thermoelectrics

## Abstract

High entropy metal chalcogenides are an emergent class of materials that have shown exceptional promise in applications such as energy storage, catalysis, and thermoelectric energy conversion. However, the stability of these materials to factors other than temperature are as yet unknown. Here we set out to assess the stability of the high entropy metal sulfide (MnFeCuAgZnCd)S with high pressure (up to 9 GPa), compared to an enthalpically stabilised Ag_3_CuS_2_, and a *quasi*-stable (MnFeZnCd)S. Compression and pressure-annealing of (MnFeCuAgZnCd)S showed diffusion-controlled time and pressure dependent exsolution of jalpaite (Ag_3_CuS_2_) from the bulk. Bulk materials characterisation found minor phase impurities and possible elemental localisations in (MnFeCuAgZnCd)S prior to pressure-annealing. To gain deeper understanding of the material pre- and post-pressure annealing at the nanoscale an advanced technique was used which combined machine learning, unsupervised clustering analysis of STEM-EDX mapping with scanning precession electron diffraction (SPED), which revealed a chemically distinct post-pressure annealed jalpaite exsolved from (MnFeCuAgZnCd)S.

## Abstract (web summary)

Although high-entropy metal chalcogenides are attractive candidates for energy conversion and catalysis, their stability under high pressure is not well understood. Here, a combination of electron microscopy, elemental analysis and electron diffraction with machine learning shows that compression and pressure-annealing of the (MnFeCuAgZnCd)S wurzite leads to exsolution of a chemically distinct jalpaite.

## Introduction

High entropy (HE) materials^1,2^ (multicomponent materials with at least 5 or more elements in a single lattice or sub-lattice) are an emergent, synthetic class of materials that are stabilised into a single crystalline phase by entropy, in competition against enthalpy^3,4^. Entropic stabilisation is also observed is some natural materials^5–7^, which have, at some time, been close to thermodynamic equilibrium^8^. In addition, many naturally occurring minerals are enthalpically stable but can have a high number of possible components on a single cation site (e.g. garnets, tourmalines, plagioclases)^9^, and consequently may contain reasonable entropy. Unlike synthetic HE materials, natural materials very rarely have equal proportions of numerous principal cations and only very infrequently contain elements that are not enthalpically achievable within the lattice^10^. Synthetic HE materials can also be exploited to trap unfavourable cations in a lattice and create metastable material phases^11,12^.

When subject to high pressure, most materials undergo phase transitions to other denser structures, many of which are not accessible at ambient pressure^13^. Some (but not all) of these high-pressure structures are recoverable to ambient pressure as metastable phases (e.g. diamonds). The effect of pressure on medium and high entropy alloys^14–17^, and high entropy metal oxides^18^ shows similar uniquely pressure-driven phenomena. For example, a pressure induced continuous tuning of lattice distortion (bond angles) and band gap has been reported in the (Ce_0.2_La_0.2_Pr_0.2_Sm_0.2_Y_0.2_)O_2−δ_ high entropy metal oxide^18^. In situ high-pressure X-ray diffraction has revealed a polymorphic transition from a face-centred-cubic structure to a hexagonal close-packed structure in the CoCrFeMnNi high-entropy alloy, at *ca*. 25 GPa^15^.

HE materials have been stabilised with high numbers of principal elements, for example the octonary (PtPdCoNiFeAgCuAn)^19^ HE alloy and denary (LaCaSrBaFeMnCoRuPdIr)O^20^ HE metal oxide, The large numbers of elements causes significant complexity within these materials that cannot be sufficiently characterised with bulk characterisation techniques such as powder X-ray diffraction (pXRD) and scanning electron microscopy coupled to energy dispersive X-ray spectroscopy (SEM-EDX)^4,21^. Of these, metal chalcogenides are an emergent class of high entropy materials^22^ that have shown great promise in energy storage^21,23^, electrocatalysis^11,24–26^, thermoelectric energy conversion^27–29^, and as superconductors^30,31^. Previous studies have shown that high entropy metal sulfides can be stabilised with a wurtzite structure^32^. Wurtzite is a naturally occurring mineral form of (ZnFe)S with a hexagonal space group $P{6}_{3}{mc}$. Other sulfides that adopt the wurtzite structure include greenockite [CdS] and rambergite [MnS]. Without iron, zinc sulfide naturally forms sphalerite (ZnS, $F4\bar{3}m$), which has common impurities that include Fe, Cd, and Mn. Synthetically, wurtzite structures of alloyed (Zn_*x*_Cd_1-*x*_)S^33,34^, (Cd_1-*x*_Mn_*x*_)S^35^, and Mn and Fe doping of ZnS^36–38^ and CdS^39,40^ have all been reported. Neither copper nor silver naturally form wurtzite structured sulfides. Although, copper has been reported in wurtzite structured copper tin sulfides^41^, copper indium sulfide^42^, and high entropy (ZnCoCuInGa)S metal sulfide nanoparticles^32^. To the best of our knowledge there are no reports of any wurtzite structured sulfides containing silver, although silver iodide is a wurtzite structured material^43^. To date, the only reports combining high entropy metal chalcogenides and high pressure has been reported on metal tellurides, as either a synthetic step, which undergo an initial thermal annealing step (under vacuum), followed by further thermal annealing at high pressure (3 GPa) for 30 min, forming pellets^30,31^. (AgInSnPbBi)Te has been reported to undergo a phase transition from rock salt to CsCl structures at pressures >17 GPa. This pressure-phase relationship has been reported to have an impact on the onset superconducting temperature (*T*_c_) of these materials, with higher *T*_c_ values found with increasing pressure^44,45^.

Although many simple sulfides are known to exist^46^, relatively few have been studied at high pressure and pressure-temperature (PT) phase diagrams are often not known. Simple iron sulfides have been studied at pressures up to 20 GPa (e.g. FeS)^47,48^ or even 300 GPa (e.g. Fe_2_S)^49^, to understand planetary cores. But most metal sulfides’ PT phase diagrams are only known to only a few GPa (e.g. ZnS^50^, the CdS-MnS binary)^51^, or have only been investigated at ambient temperature (e.g. Ag_2_S)^52,53^ or have not yet been studied. To date, no investigation as to the effect of high pressure on the stability of multi-principal cation metal sulfides has yet been undertaken.

Therefore, in this study we set out to use high pressure (up to 9 GPa) to test the stability of three low, medium, and high entropy metal sulfides in the (Mn-Fe-Cu-Ag-Zn-Cd)S system: specifically high entropy (MnFeCuAgZnCd)S, medium entropy (MnFeZnCd)S, and low entropy Ag_3_CuS_2_. Comprehensive characterisation of these materials is extremely challenging due to their complexity. Typical phase identification using powder X-ray diffraction (pXRD) was complimented by microscale (scanning electron microscopy, SEM) and nanoscale (scanning transmission electron microscopy, STEM) imaging and energy dispersive X-ray (EDX) spectroscopy for elemental analysis. The STEM-EDX mapping spectra were processed using an unsupervised clustering approach to assess elemental homogenisation. Scanning precession electron diffraction (SPED) was also measured within the same region to analyse nanoscale phase analysis. With this advanced characterisation we therefore report the first example of combined nanoscale-level stability, elemental, and phase analysis in these highly complex, emergent materials.

## Results

**Fig. 1:**
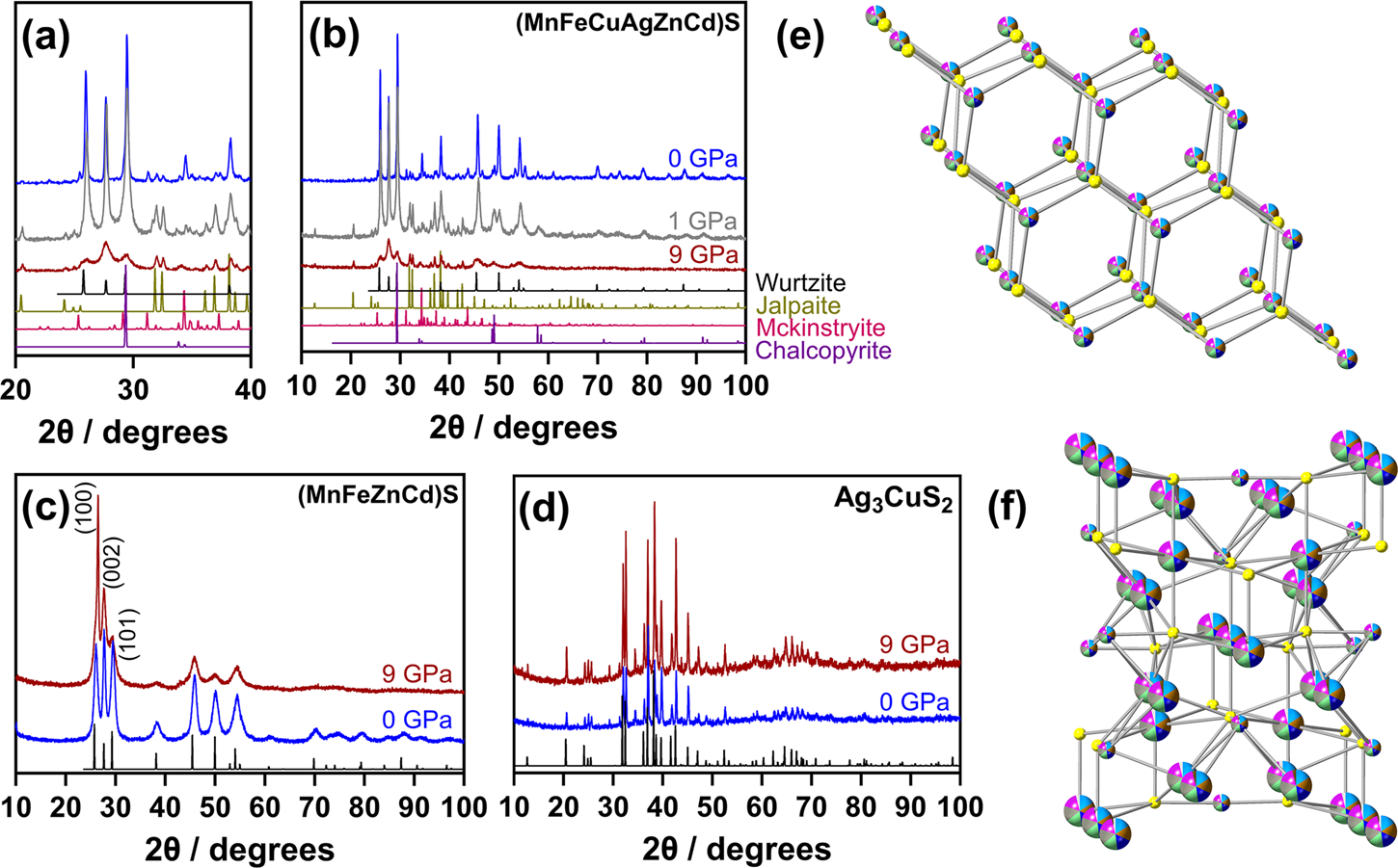
Powder X-ray diffraction and crystal structures of samples in this study. **a**, **b** (MnFeCuAgZnCd)S, **c** (MnFeZnCd)S and **d** Ag_3_CuS_2_ both before (blue) and after (red) subjection to 9 GPa of pressure-annealing. Also shown in (**a**, **b**) in grey is the pXRD pattern when (MnFeCuAgZnCd)S is subjected to 1 GPa of pressure-annealing. Model patterns correspond to wurtzite (ICSD: 67453) (**a**–**c**), jalpaite (ICSD: 67526) (**a**, **b**, **d**), McKinstyrite (ICSD: 169681) (**a**, **b**) and chalcopyrite (ICSD: 2516) (**a**, **b**). Supplementary Fig. S1 shows the same Figure with miller indices indicated. **e** and **f** are crystal structures of the high entropy (**e**) wurtzite (ICSD: 67453) and (**f**) jalpaite (ICSD: 67526) lattices investigated in this study, with multicoloured spheres representing the random arrangements of metals in the cationic positions.

Three metal sulfides ((MnFeCuAgZnCd)S, (MnFeZnCd)S, and Ag_3_CuS_2_) were synthesised *via* a solventless thermolysis, single source precursor approach previously reported^10,11^ and detailed in the SI. The resulting metal sulfide powders were characterised by powder X-ray diffraction (pXRD), Raman spectroscopy, and scanning electron microscopy (SEM) coupled to energy dispersive X-ray (EDX) spectroscopy and are shown in Fig. 1 and Supplementary Figs. S1–S5. The crystalline phases present were determined through analysis of the pXRD patterns (Fig. 1) and were found to compose of predominantly hexagonal wurtzite (ICSD: 67453) for both (MnFeCuAgZnCd)S and (MnFeZnCd)S, and jalpaite (ICSD: 67526) for Ag_3_CuS_2_. However, there were some minor impurities present in (MnFeCuAgZnCd)S that could be indexed to jalpaite, Mckinstryite (ICSD: 169681), and possibly chalcopyrite (ICSD: 2516), although there is significant overlap between the peaks of standard chalcopyrite and wurtzite, as these crystal structures are simply different stacking sequences of the same metal-sulfur units^54,55^. Raman spectroscopy (Supplementary Fig. S2) was also used to confirm present phases, with SEM-EDX used to analyse microscale elemental homogeneity of the resulting products, both discussed in Supplementary Figs. S2–S5.

**Fig. 2:**
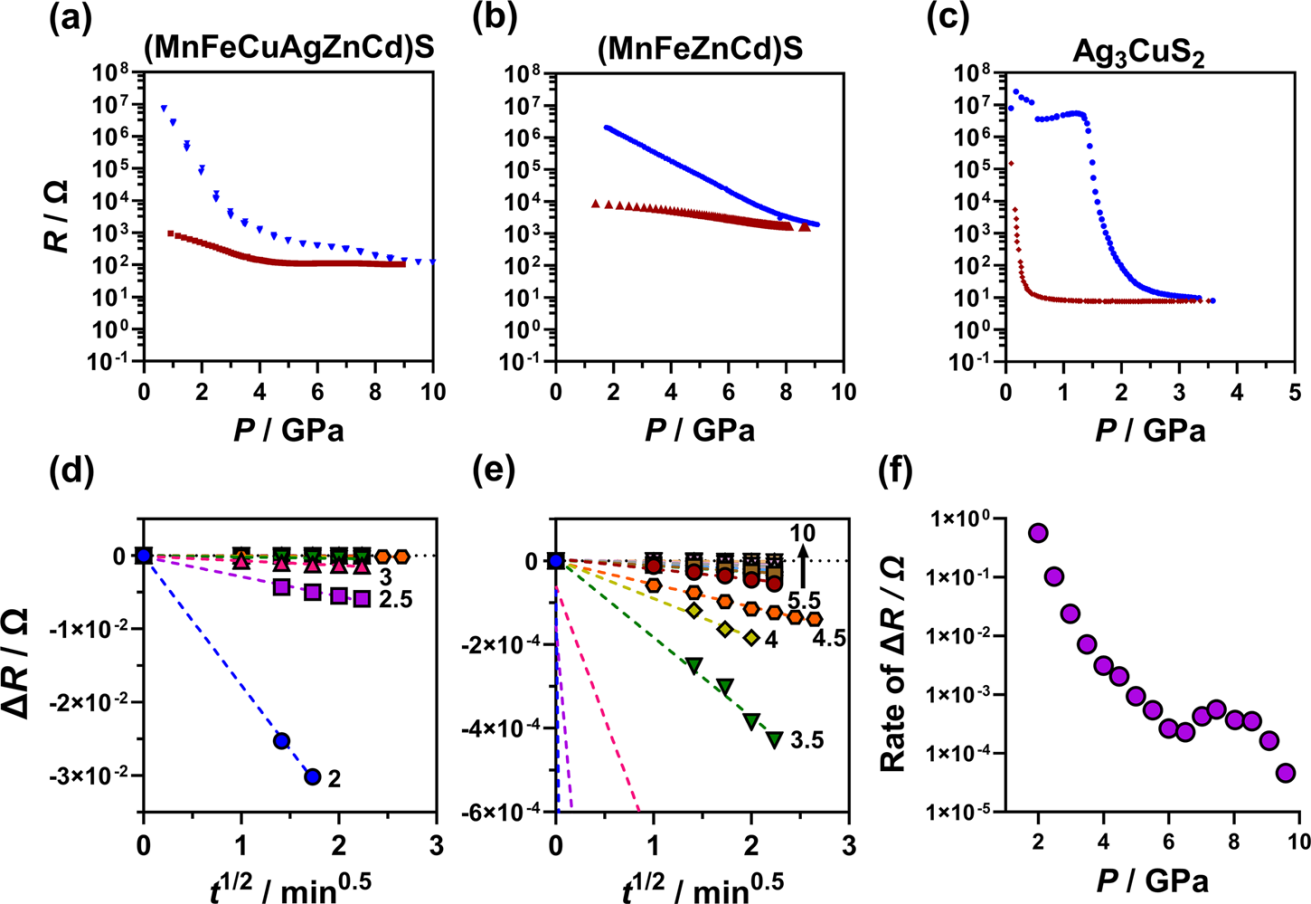
Resistance data of Ag_3_CuS_2_, (MnFeZnCd)S, and (MnFeCuAgZnCd)S with pressure. Panels a-c show resistance during increasing pressure (blue) and decreasing pressure (red) for the three compositions in this study. The values in panels (**d**, **e**) show the change in resitance (R) against time in (MnFeCuAgZnCd)S at pressures annotated. **f** shows the rate of change of *R* at each pressure. Supplementary Fig. S6 shows pressure of Ag_3_CuS_2_ up to 9 GPa with no further change in resistance.

The stability of the enthalpically stabilised Ag_3_CuS_2_, *quasi*-stable (MnFeZnCd)S, and entropically stabilised (MnFeCuAgZnCd)S was assessed by subjecting all three systems to pressures up to 9 GPa, in a multi-anvil apparatus^56^. The resistance of Ag_3_CuS_2_, (MnFeZnCd)S, and (MnFeCuAgZnCd)S were measured in situ during pressure increase and decrease. Figure 2 shows the change in resistance with both increasing pressure (blue data) and decreasing pressure (red data) for each of the samples. All three systems were found to have an initial resistance between 1 $\times$10^6^ – 1 $\times$10^7^ Ω as synthesised powders. The resistance rather than resistivity of the samples is reported because not cross-sectional area of the sample is not known at high pressure; the cross-sectional area of the pre-compressed powder is of order 1 mm^2^ and the sample is 3–4 mm in length (see methods for details). With increasing pressure, all samples exhibit a significant drop in resistance. Ag_3_CuS_2_ shows the most ‘typical’ behaviour of the three samples (Fig. 2c). During compression, there is a drop in resistance of 6 orders of magnitude at *ca*. 2 GPa. Similar drastic changes in resistance are observed in GaP and GaS during reversible metal-insulator transitions^57–60^. During decompression, the resistance of Ag_3_CuS_2_ increases suddenly towards the initial value. It occurs at apparently lower pressure than during compression because of uncalibrated hysteresis in the force-pressure relationship of the experimental apparatus^57^. Similar behaviour is observed during force-pressure calibration experiments using ZnS, GaP, or GaAs, all of which undergo insulator-metal transitions at high pressures (Supplementary Fig. S6)^57,61^. Thus, we interpret this change in resistance as a ‘typical’ low enthalpy, low-entropy, metal-insulator type transition, whereby both pXRD (Fig. 1d) and Raman spectroscopy (Supplementary Fig. S2) analysis showed that Ag_3_CuS_2_ was completely recoverable after pressure annealing. To the best of our knowledge there are no previous reports of high-pressure phase transitions in Ag_3_CuS_2_ with which to corroborate this.

The two entropy stabilised phases behave very differently. The resistivity of (MnFeZnCd)S shows a log-linear relationship with pressure that only partially recovers during decompression (Fig. 2b). The conductivity of the crystal orientation rearranged material during/after pressure is higher (i.e. consistently lower resistance) than the initial material. This is indicative of an unrecoverable change in the sample at high pressure, whereby a significant change in pXRD diffraction peak intensity was observed (Fig. 1c) in relative intensity and sharpness of the ($110$) peak, and a decrease in the ($01\bar{1}$), and ($01\bar{3}$) peaks, without any obvious change in elemental homogenisation. This could reflect either a change in crystal preferred orientation or, more likely, changes in cation site ordering within the same crystal structure.

(MnFeCuAgZnCd)S undergoes the most unusual behaviour. The resistance of the (MnFeCuAgZnCd)S decreases non-linearly with pressure, showing a distinct kink in resistance at *ca*. 3 GPa during compression. Unlike resistance experiments on calibration materials (such as GaP, GaAs), (MnFeCuAgZnCd)S does not return to its original resistance after the removal of pressure, instead remaining low. During compression of this experiment, the compression was paused every ~0.5 GPa and the resistance of the sample measured as a function of time, for up to 5 min (Fig. 2d–f). The decrease in resistance change was approximately linear with t^1/2^. The rate of resistance change (Δ*R*) decreases at higher pressure and with sample resistance (Fig. 2f). This is abnormal behaviour compared to resistances measured in other materials (e.g. Bi, ZnS, GaP and the other sulfides in this study) where the time scale of resistance change is of the order of the pressure change. The Δ*R* was not observed in Ag_3_CuS_2_, nor (MnFeZnCd)S. (MnFeCuAgZnCd)S also shows the most significant changes in pXRD after pressure-annealing. Figure 1a, b shows that the pXRD peaks relating to the wurtzite structure are significantly lower in intensity and significantly broader after the material was subjected to high pressure. This phenomenon is common in nanomaterials, where peak broadening is attributable to a decrease in crystallite size (i.e. Scheerer broadening)^62^, which wasn’t observed in a sample that was only subjected to 1 GPa (Fig. 1a, b). Peak broadening could also be due to disruption in the crystalline lattice, for example with the presence of significant quantities of vacancies^63^. The peaks indexable to jalpaite were also found to be significantly more intense in the pressed samples (even after only 1 GPa), compared to the as synthesised sample.

Electrical resistance changes with time at each held pressure (Fig. 2) is evidence of (MnFeCuAgZnCd)S undergoing a continuous reaction^64^, to produce jalpaite, as observed in Fig. 1. Because the changes in electrical resistance are linear in *t*^1/2^, it seems likely that the process is diffusion controlled^64^, rather than alternative mechanisms. In this case the Ag and Cu (and possibly Cd) cations diffuse out of the Wurtzite lattice, forming Jalpaite phased material observed in the STEM analysis *vide infra* and pXRD analysis *vide supra*. The nascent jalpaite has sharp diffraction peaks, indicating the crystallite size potentially reaches the bulk limit. We therefore may also tentatively conclude that the diffusion of Au and Cu cations occurs on length scales significantly greater than that of the unit cell based on these data.

**Fig. 3:**
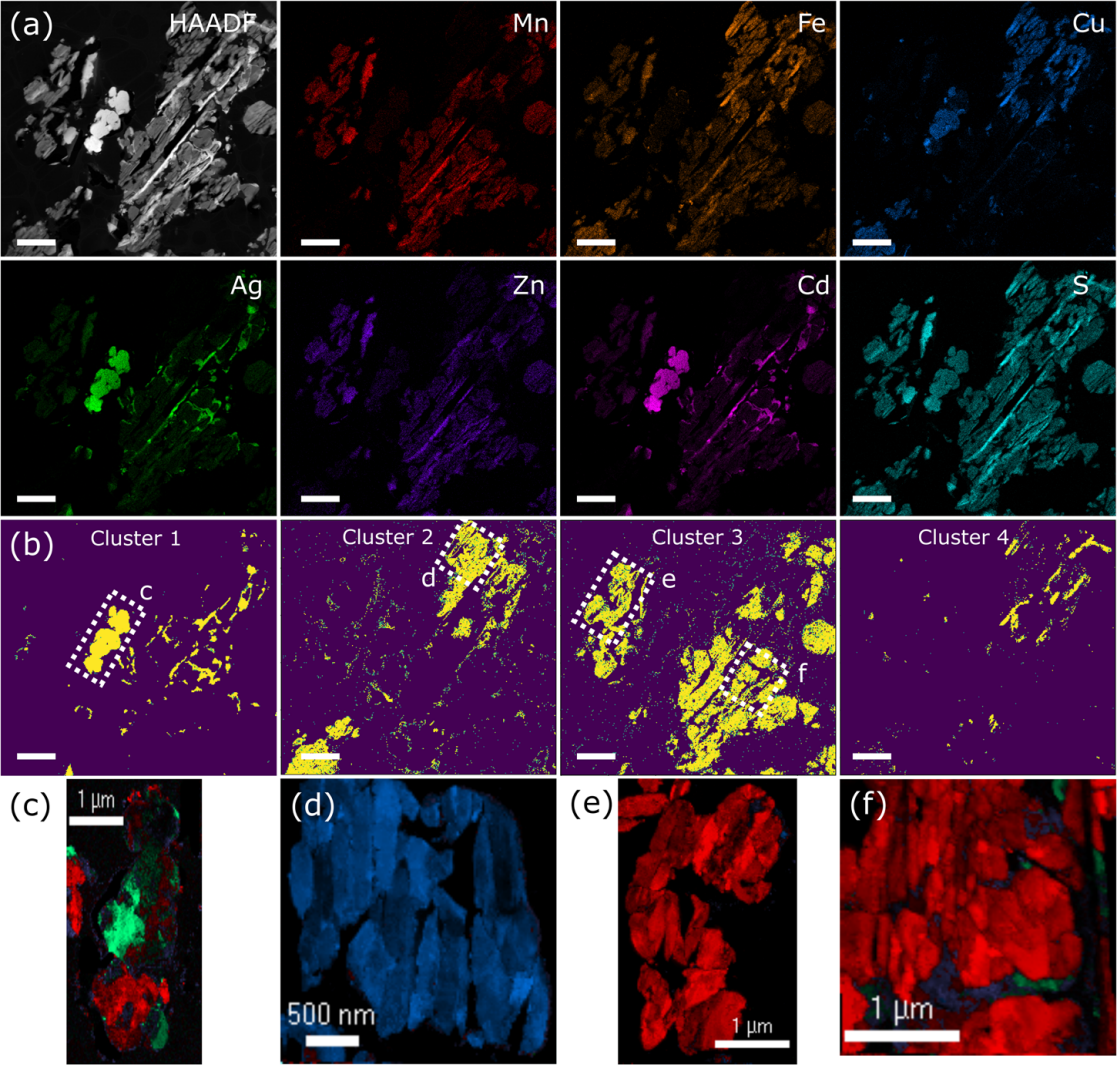
STEM-EDX of the (MnFeCuAgZnCd)S, prior to pressure annealing. **a** HAADF-STEM-EDX maps; the scale bar represents 2 µm. **b** STEM-EDX cluster membership maps for the four distinct compositions identified in the scan. The rectangular regions outlined were re-analysed using SPED and phase mapping (ACOM) in (**c**–**f**). In these images, red pixels indicate the diffraction pattern was matched to wurtzite, blue indicate chalcopyrite, green indicate jalpaite and purple indicate Mckinseyite. The colour intensity indicates the pattern cross-correlation score so vacuum pixels and grain boundaries appear dark.

**Fig. 4:**
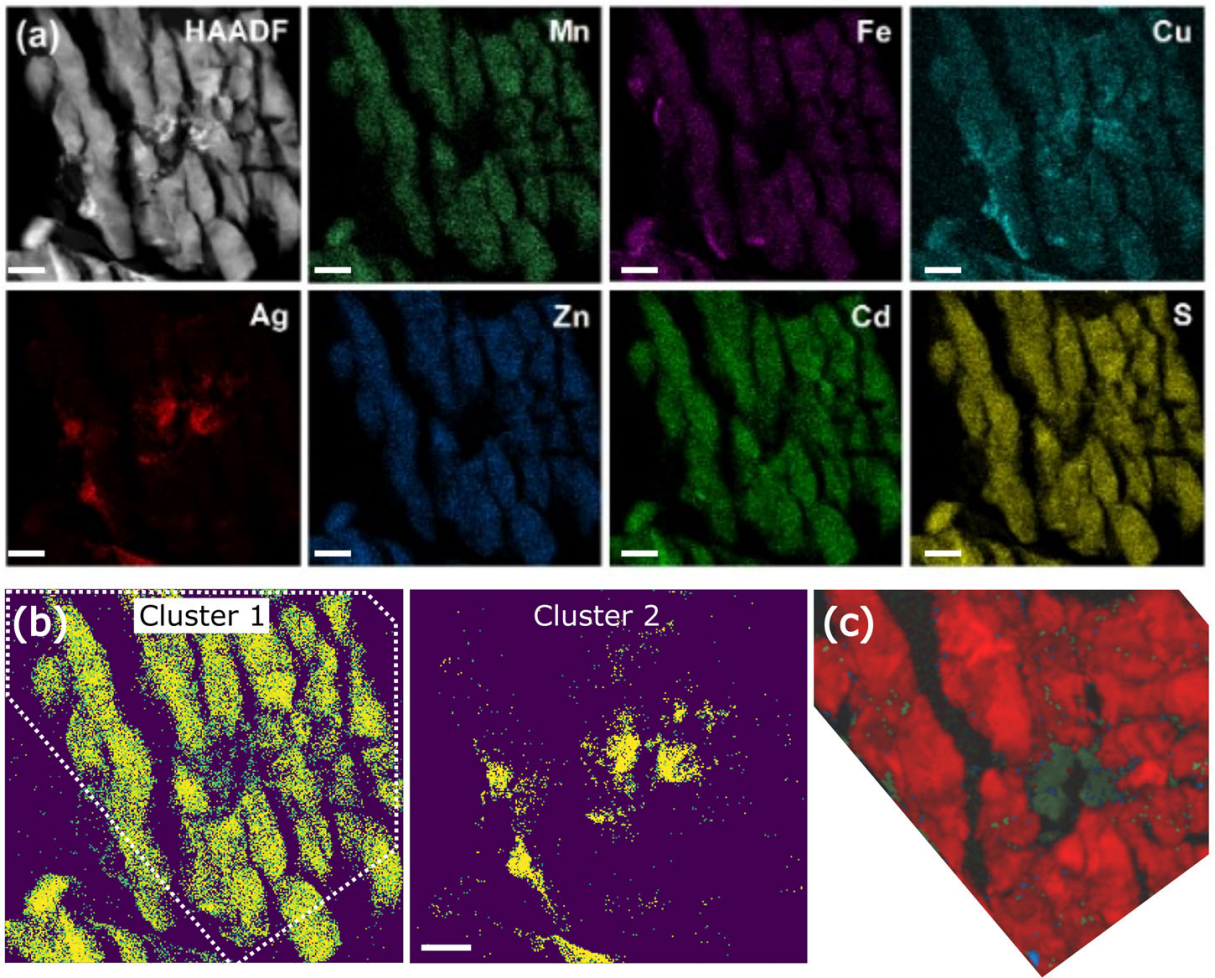
STEM-EDX of the (MnFeCuAgZnCd)S, Region 1, after pressure annealing at 9 GPa. **a** STEM-EDX map; scale bar represents 200 nm. **b** STEM-EDX cluster maps for the two major compositions. **c** shows the phase mapping for the region marked by the dotted line in (**b**) red pixels indicate a match to wurtzite and green indicate jalpaite.

**Fig. 5:**
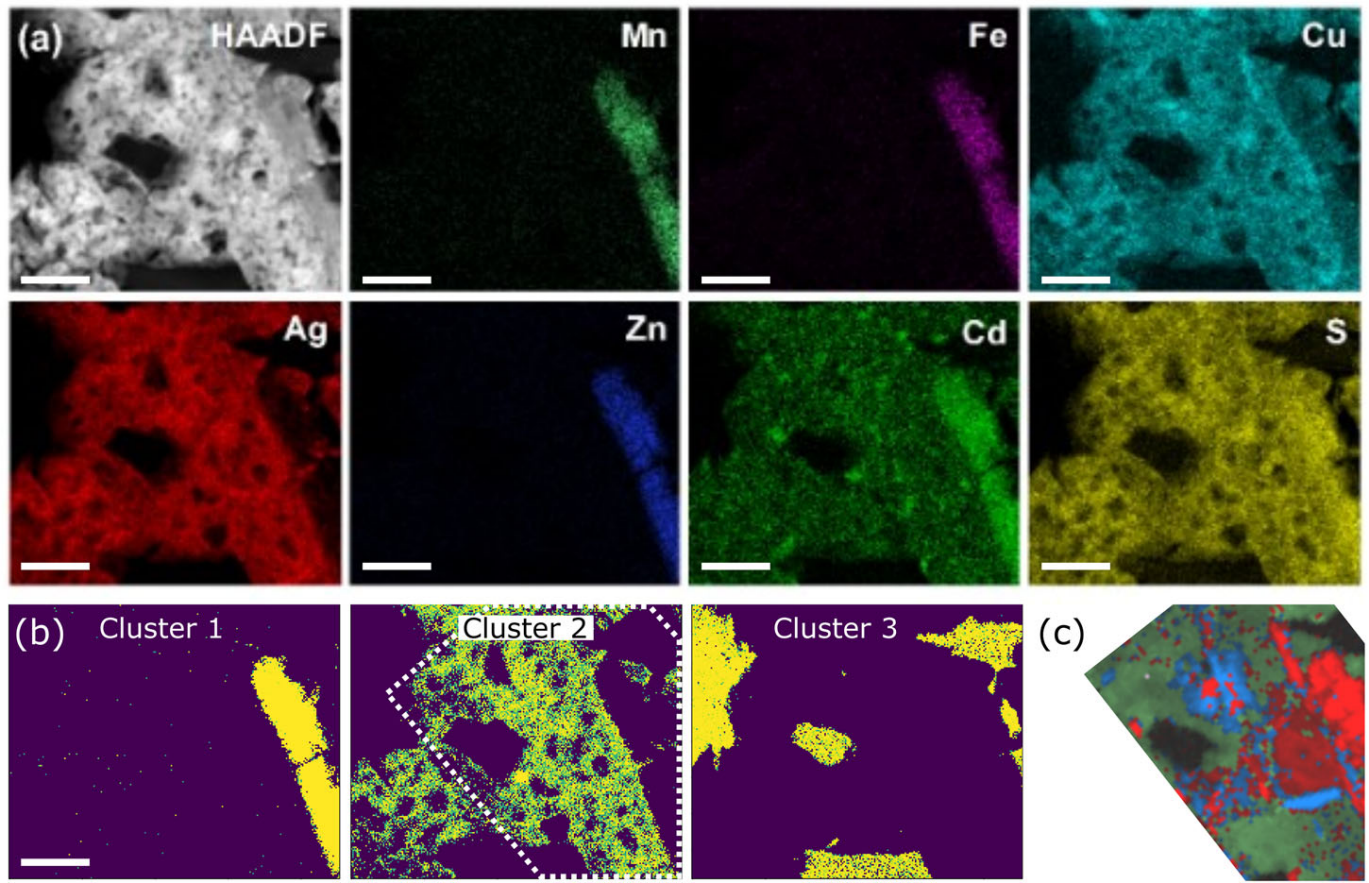
STEM-EDX of the (MnFeCuAgZnCd)S, Region 2, after pressure annealing at 9 GPa. **a** STEM-EDX map; scale bar represents 200 nm. **b** shows STEM-EDX cluster maps for the three major compositions in the region, **c** shows the phase maps for the region indicated by the dotted line in (**b**). For the phase map, red pixels indicate a match to wurtzite, blue indicates chalcopyrite and green indicates jalpaite.

In order to further analyse the exsolution of jalpaite, apparent from the pXRD and in situ resistance analysis, we undertook advanced, nanoscale characterisation using scanning transmission electron microscopy (STEM-EDX) mapping combined with scanning precessional electron diffraction (SPED, selected diffraction data with fitting shown in Supplementary Fig. S14). STEM-EDX maps were measured on (MnFeCuAgZnCd)S both before (Fig. 3a), and after (Figs. 4a and 5a) pressure annealing. This imaging analysis indicates that the 6 major metallic elements are not homogenously distributed throughout the HE material prior to pressure annealing, but instead tend to form exclusive regions that are richer in one of (Mn-Fe-Zn-Cd)S, (Fe-Cu)S, or (Ag-Cu-Cd)S. A machine learning, unsupervised clustering (see methods section for full details) approach to analyse the STEM-EDX map in the same area as SPED was undertaken. Rather than the traditional approach of correlating pairs of elements in the spectra, this approach groups together spectra with similar characteristic peaks and so provides an overview of the multi-element correlations occurring in the sample. This approach found 4 distinct compositions within the scanned region (Fig. 3b). Quantitative analysis of metal stoichiometry (Sulfur stoichiometry was not determined by this process given the difficulty of preparing accurate quantification standards.) (in at. %) was undertaken on selected (indicated in Fig. 3b) regions of interest, (shown in Supplementary Fig. S7), with the results shown in Table 1. Cluster 1 of (MnFeCuAgZnCd)S is composed predominantly of silver, copper, and cadmium sulfide, with the particle indexed in Fig. 3c. A large amount of the region is comprised of jalpaite-structured material (shown in green), though there are clearly grains of silver-rich wurtzite also present (shown in red). Cluster 2 is almost entirely comprised of iron and copper sulfide, with the diffraction analysis (shown in Fig. 3d) almost entirely indexed to chalcopyrite (shown in blue). Clusters 3 and 4 comprise a mixture of manganese, iron, zinc, and cadmium, with the minority phase also containing copper. The phase maps for these regions (Fig. 3e, f) indicate a majority phase of wurtzite, though there is some overlap with cluster 1 in this region, indicating the presence of minority jalpaite structured materials in these areas.

**Table 1:** Quantitative stoichiometry of the metals determined from the cluster-average EDX spectrum (in atomic %), where cluster numbers match those given in Fig. 3b with phase identification from Fig. 3c–f

	Cluster 1 (majority jalapite)	Cluster 2 (majority chalcopyrite)	Cluster 3 (majority wurtzite)	Cluster 4 (majority wurtzite)
Ag	47.1 ± 2.5	0.8 ± 0.1	3.2 ± 0.1	4.0 ± 0.2
Cd	31.7 ± 1.7	0.8 ± 0.1	17.2 ± 0.3	13.5 ± 0.4
Cu	13.4 ± 0.9	46.2 ± 1.2	2.6 ± 0.1	13.7 ± 0.7
Fe	3.8 ± 0.3	50.3 ± 1.2	24.2 ± 0.6	30.5 ± 1.0
Mn	2.4 ± 0.2	1.0 ± 0.1	29.7 ± 0.7	21.4 ± 0.7
Zn	1.6 ± 0.2	1.0 ± 0.1	22.8 ± 0.5	17.0 ± 0.6

This combined analysis shows that silver-rich regions are mostly, but not entirely indexed as jalpaite, though there are clearly wurtzite regions that also contain silver (indicating that some septenary, high entropy material is present). The wurtzite and jalpaite structures appear to be very close in overall free energy, given their coexistence in the silver-rich cluster, as such we cannot exclude additional jalpaite formation as a result of mechanical energy applied to the sample during preparation for TEM analysis. The preference for regions rich in both iron and copper to form chalcopyrite is not unexpected but provides further evidence for enthalpic factors being potentially significant in the formation of the HE material.

Figures 4 and 5 show the same analysis on two differing areas of (MnFeCuAgZnCd)S post-pressure annealing. Region 1 (Fig. 4) was found to contain only two distinct compositions, with their quantitative composition given in Table 2. The SPED analysis (Fig. 4c) shows the majority of the region to be wurtzite, with an individual grain size present of 10–100 nm (*c.f*. the ~μm grains in Fig. 3). There is also a small jalpaite inclusion found here. The wurtzite phase is comprised of all of the metals in the HE material (except silver), which is not dissimilar to the typical stoichiometry seen in the uncompressed material. This analysis also indicates that some jalpaite (Cluster 2) appears to have transformed from the parent wurtzite (making it a secondary jalpaite) that is less chemically distinct from the parent (MnFeCuAgZnCd)S compared to the ‘primary’ jalpaite observed prior to pressure-annealing (Table 2).

**Table 2:** Cluster average stoichiometry for the metal distributions for the clusters indicated in Fig. 4b (atomic %)

	Cluster 1 (wurtzite)	Cluster 2 (jalpaite)
Ag	3.5 ± 0.2	12.4 ± 0.7
Cd	17.0 ± 0.5	19.1 ± 1.0
Cu	25.4 ± 0.8	29.6 ± 1.6
Fe	15.4 ± 0.5	12.4 ± 0.8
Mn	19.1 ± 0.6	12.8 ± 0.8
Zn	19.7 ± 0.6	13.6 ± 0.9

Region 2 of the pressure-annealed (MnFeCuAgZnCd)S shows three major compositions, shown in Fig. 5, with the first two cluster average stoichiometries given in Table 3 and the SPED analysis shown in Fig. 5c. There is a single linear feature shown in cluster 1 that is mostly indexed to wurtzite, and which contains all 6 metals (including silver). The remainder of the region is almost all represented by cluster 2, which is rich in silver, cadmium, and copper, suggesting jalpaite. However, it was not possible to conclusively index the diffraction patterns. The suggestion is that the region is predominantly jalpaite (as expected from the composition) but with extremely small (of the order of 10 s of nm) grains of retained wurtzite and chalcopyrite. However, this could also be the result of overlapping projections of multiple grains of any of the phases along the beam path, which makes unambiguous indexing the patterns more difficult. Either way, the presence of such a fine-scale microstructure would make absolute diffraction analysis difficult in many regions and supports the broadening of the peaks in the pXRD analysis, and the requirement of the advanced clustered STEM-EDX, SPED nanoscale analysis. The final microstructure component identified by cluster 3 is a carbon rich phase, possibly a remnant of the embedding resin used for sectioning and the stoichiometry is not included here.

**Table 3:** Cluster average stoichiometry for the metals in region 2 (in atomic %)

	Cluster 1	Cluster 2
Ag	12.6 ± 0.6	29.4 ± 1.5
Cd	17.9 ± 0.8	22.1 ± 1.1
Cu	31.9 ± 1.4	24.6 ± 1.3
Fe	10.4 ± 0.6	0.3 ± 0.1
Mn	12.8 ± 0.7	0.1 ± 0.1
Zn	14.5 ± 0.8	0.3 ± 0.1

Cluster numbers correspond to the regions indicated in Fig. 5b.

Two further regions of post-pressurised (MnFeCuAgZnCd)S were analysed and are shown in Supplementary Figs. S8 and S9 and Supplementary Tables S4 and S5, which showed the same trend of element rich samples post-pressure annealing and cemented the utility of this method. (MnFeZnCd)S in the absence of Cu-Ag was also measured using combined STEM-EDX, SPED analysis and is shown in Supplementary Figs. S10–S13 and Supplementary Tables S6–S9. This analysis showed no further formation of phases, as also evidenced by the lack of further peaks in the pXRD pattern. There also appeared to be no preferential grain ordering on the scale observed ( ~ 10 s of nms). We postulate that the change in pXRD peak intensity (Fig. 1c) could be due to a cation reordering on the unit cell (~Å) level not observable here, although analysis of these materials indicate no significant cation reordering on the ~10 s of nms scale, which does indicate no significant diffusion of cations under pressure. One final discovery in this analysis was the presence of pyrrhotite (Fe_7_S_8_, ICSD: 37166) in the pre-pressurised system. This was not evidenced in the pXRD pattern and demonstrates the efficacy of this nanoscale analysis.

The summary of the structural analysis of (MnFeCuAgZnCd)S seems to be that during the compression stage, there is a considerable change in the silver distribution in the material, with secondary jalpaite forming from the parent wurtzite (containing more elements than the initial jalpaite). This transformation also leading to an overall reduction in the grain size in the material as well as a far more disrupted nanostructure.

## Discussion

High pressure was used as a method of interrogating the relative stability of entropically stabilised (MnFeCuAgZnCd)S, *quasi*-stable (MnFeZnCd)S and enthalpically stabilised Ag_3_CuS_2_ materials. Powder XRD of the pressure annealed (MnFeCuAgZnCd)S showed that at high pressure it had exsoluted a jalpaite structured phase (Ag_3_CuS_2_) and that the remaining wurtzite structured phase was extremely disrupted. The disruption could be due to the formation of significant quantities of vacancies, or, less likely, the significant decrease in crystallite size. Significant vacancy formation is likely because of the change in cation-sulfur ratio between the wurtzite and jalpaite structures. The (MnFeZnCd)S and Ag_3_CuS_2_, both retained their original structure, indicating that the (MnFeCuAgZnCd)S is significantly less stable under pressure.

To gain insight into the nanoscale phase and elemental localisation of (MnFeCuAgZnCd)S before and after pressure-annealing, a combined STEM-EDX unsupervised clustering SPED method was used. This analysis found that minority Ag_3_CuS_2_ jalpaite and CuFeS_2_ chalcopyrite were present in the original (MnFeCuAgZnCd)S, but further jalpaite was exsoluted from (MnFeCuAgZnCd)S after pressure-annealing, which retained more of the original elements (i.e Mn, Fe, Zn, Cd) than the jalpaite present prior to pressure annealing. This insight was only possible using this advanced characterisation technique which – for the first time – allows both nanoscale elemental and phase analysis on these highly complex materials. In situ resistance measurements of pressurising (MnFeCuAgZnCd)S also indicated that the rate of exsolution of jalpaite occurred on a measurable timescale (*i.e*. mins), which is remarkable for a thermodynamically driven process in the absence of external thermal energy and implies that the chemical potential of the original material is very high.

As shown in previous studies, pressure has a similar effect on high entropy phases to its effects on enthalpically stabilised phases. The exsolution of jalpaite at such a high rate without external thermal energy is indicative that there is a significant thermodynamic driving force for this reaction. As Ag can neither be found naturally or synthetically in wurtzite structured sulfides, it is likely to have a high dissolution energy within (MnFeCuAgZnCd)S, making Ag unfavourable in this structure and when forced into it giving high internal energy ($U$) and chemical potential. Although CuFeS_2_ was found to be greater than either Fe_7_S_8_, FeS, Cu_2_S or Cu_1.8_S, and other ternary metal sulfides were not calculated. In contrast, the newly formed jalpaite-Ag_3_CuS_2_ is likely to have a low internal energy, supported by its natural occurrence and it being the favourable phase for Cu over (MnFeCuAgZnCd)S.

A large $\Delta U$ of formation feeds into the enthalpy of formation of the jalpaite. This is because the reaction enthalpy ($\varDelta H$) in the Gibbs (or Helmholtz) free energy relationship implicitly contains a pressure ($P$) term: namely: $\Delta H=\,\Delta U+P\Delta V$, where $U$ in the internal energy and $\varDelta V$ the volume change of reaction. An expanded form of the usual Gibbs free energy ($G$) of a reaction is:

1 $$\Delta G=\Delta U+P\Delta V-T\Delta S$$

Where $T$ is the thermodynamic temperature and $\varDelta S$ is the change in entropy of reaction.

Jalpaite is denser than wurtzite (4–5 g cm^−3^ for (FeZn)S and CdS and 6.8 g cm^−3^ for Ag_3_CuS_2_). Thus, at high pressure, the $P\varDelta V$ term is negative and seems sufficient to drive this exsolution. However, the formation of significant quantities of vacancies forming jalpaite (2:1 metal to sulfur) from wurtzite (1:1 metal to sulfur) would normally lead to a disadvantageous $\Delta U$ in this reaction, but it could be that the $\Delta U$_formation_ of Ag in the structure is higher than that of vacancy formation. Exsolution of Ag to leave vacancies could therefore give a negative $\Delta U$. It is not possible to determine the sign of $\Delta U$ here. As jalpaite is exsoluted retaining further elements from (MnFeCuAgZnCd)S, it is expected that $\Delta S$ remains relatively high during this reaction, without a significant entropic cost. It is clear though that the $P\Delta V$ term is sufficiently large to drive the reaction, even in the absence of external thermal energy.

The change in chemistry of jalpaite formation implies significant diffusion in the samples. The chemical mapping analysis suggests significant cation diffusion is occurring, and we observe clusters of jalpaite that are significantly larger than 200 nm, with residual areas of wurtzite that are *ca*. 200 nm in maximum dimension. Diffusion distance ($x$) is^64^:

$$x= \root 2 \of {{Dt}}$$

where $t$ is time and $D$ is the diffusivity. Taking 200 nm as the length scale of the diffusion and the time of the experiment (*ca*. 2 h), we get a first order approximation of the diffusivities for the metal cations of ~ 5 $\times 10$^−14^ cm s^−1^ in our experiment. This is a very high value for diffusivity at room temperature; it is comparable to Fe diffusion in sphalerite at 500 °C^65^ and significantly faster than Cd diffusion in CdS at 25 °C^66^. It is consistent with the ‘anomalously’ fast diffusion in defect rich, off-stoichiometric, CdS^66^. Since rates of diffusion are a function of chemical potential. The high diffusion rates of copper and silver out of the wurtzite structure to make jalpaite, support their high chemical potential and the large reduction ($\Delta U$) for the formation of jalpaite.

## Conclusions

Here we have investigated - for the first time - the pressure-stability of a high entropy metal chalcogenide (MnFeCuAgZnCd)S and compared this to the pressure stability of a *quasi*-stable (MnFeZnCd)S and enthalpically stable Ag_3_CuS_2_. In situ resistance monitoring found a remarkably high rate of reaction even in the absence of an external thermal driving force. Bulk characterisation techniques were not found to be sufficient to comprehensively characterise this apparent destruction of the original wurtzite structure and exsolution of a jalpaite-structured material. Therefore, a machine learning, unsupervised clustering approach on STEM-EDX mapping was combined with SPED on (MnFeCuAgZnCd)S both prior and post pressure-annealing. This analysis found that minority jalpaite was present prior to pressure annealing composing of mainly Ag and Cu, however, jalpaite that exsoluted from the parent wurtzite post-annealing retained high levels of Mn, Fe, Zn, and Cd. This combined advanced characterisation technique therefore allowed both nanoscale phase and elemental information for these highly complex and emergent materials. As Ag (and to a degree Cu) were originally unfavourable within the parent wurtzite structure, it is possible that marginally stable high entropy materials (such as (MnFeCuAgZnCd)S) have distinct physical behaviours over more stable HE materials due to their unique high internal and chemical energies. This methodology can be further expanded to other multicomponent and high entropy materials to assess stability, and compositional and phase of highly complex materials, even at the nanoscale.

## Supplementary information

Supplementary Material
