# Supplementary material for: Using high pressure to investigate the stability of a high entropy wurtzite structured (MnFeCuAgZnCd)S
Source: Commun Chem. 2025 Mar 5;8:65. doi: 10.1038/s42004-025-01463-9 (PMC11882918; doi:10.1038/s42004-025-01463-9)
Supplement: Supplementary file 1 — Supplementary Material [file 42004_2025_1463_MOESM1_ESM.pdf]

Supplementary information for:

Using high pressure to investigate the stability of a high entropy wurtzite structured (MnFeCuAgZnCd)S

Mark A. Buckingham,[a] Joshua J. Shea,[a] Kho Zhi Quan,[a] Pok Man Ethan Lo,[a] Joshua Swindell,[a] Weichen Xiao,[a] David J. Lewis,[a,*] Alex S. Eggeman,[a,*] and Simon A. Hunt[a,*]

[a] Department of Materials, The University of Manchester, Oxford Road, M13 9PL.

* Corresponding authors: David.lewis-4@manchester.ac.uk, alexander.eggeman@manchester.ac.uk, simon.hunt@manchester.ac.uk

Experimental

Chemicals

Sodium diethyldithiocarbamate trihydrate (Sigma Aldrich), manganese(ii) chloride tetrahydrate (Sigma Aldrich, ≥99%), iron(iii) chloride hexahydrate (≥98%, Sigma Aldrich), copper(ii) chloride (Sigma Aldrich, 97%), silver(i) diethyldithiocarbamate (Fluorochem), zinc(ii) chloride (Honeywell), cadmium(ii) nitrate tetrahydrate (Sigma Aldrich, ≥99.0%) were all purchased from UK suppliers and used without further purification.

Instrumentation

Infra-Red (IR) spectroscopy was measured on a Brucker alpha Platinum ATR FTIR instrument. Elemental analysis (EA) was undertaken on a Thermo Scientific Flash 2000 Organic Elemental Analyzer for CHN and S analyses. NMR spectroscopy was conducted on a 400 MHz Bruker AVIII HD 400. Powder X-ray diffraction was measured on a Bruker D8 Discover GIXRD Autochanger with a Cu Kα X-ray source with a wavelength of 1.541874 Å. No post-running normalisation has been undertaken on any of the pXRD patterns reported here. Indexing of diffraction patterns was undertaken using Highscore software. Raman spectroscopy was performed on a Horiba LabRAM instrument using a 488 nm wavelength laser with a 50× objective. Scanning electron microscopy coupled to energy dispersive X-ray spectroscopy was performed on a Quanta 650, equipped with a field emission gun at an accelerating voltage of 20 eV. TEM experiments were performed on a Thermo-Fisher Talos F200X microscope operated at 200kV. EDX measurements were performed with a Super-X SDD detector (0.8Sr solid collection angle). SPED experiments were conducted using a nanomegas Digistar precession control unit with diffraction patterns recorded on a Quantum Detectors MerlinEM quad hybrid-pixel detector. Experimental patterns were compared against libraries of patterns

simulated from publically available cif files for the wurtzite,[7] chalcopyrite,[8] and Jalaite[9] structures.

Synthesis of metal dithiocarbamate precursors

Synthesis of Mn(diethydithiocarbamate)$_3$ ([Mn(DTC)$_3$])

Synthesis of [Mn(DTC)$_3$] was undertaken following an adapted previous procedure.[1] Separately, $MnCl_2$ (1.98 g, 10 mmol) was dissolved in 50 mL of methanol and NaDTC (6.76 g, 30 mmol) was dissolved in 150 mL of methanol, both with stirring. The solution of $MnCl_2$ was gradually added to the solution of NaDTC while under constant stirring (note that the Mn(ii) readily oxidises to Mn(iii) upon formation of Mn(DTC)$_2$.[2] This was left for 2 hours, and the resultant precipitate collected under vacuum filtration, analysis of this product was found to be impure so a purification step was required by dissolving all impure material in 20 mL of dichloromethane with stirring for a short period, followed by the rapid addition of 10 mL ethanol. Stirring was continued for a couple of minutes, followed by a period of rest without stirring for an equivalent amount of time. The black filtrate was dried under vacuum overnight and the resultant dry powder used in subsequent investigations. Yield 54%. Elemental analysis found (expected for Mn($C_{15}H_{30}N_3S_6$) · 0.5 H_2O. C: 35.3 (35.4), H: 6.4 (5.9), N: 8.1 (8.3), S: 37.1 (37.7). IR (cm^{-1}): 571, 596, 782, 844, 912, 988, 1069, 1094, 1143, 1205, 1266, 1295, 1348, 1377, 1424, 1440, 1457, 1487, 1509, 2865, 2926, 2971. m/z: 499.0. μ (μ_{eff}): 5.07 (4.90) BM.

Synthesis of Fe(diethyldithiocarbamate)$_3$ ([Fe(DTC)$_3$])

Synthesis of [Fe(DTC)$_3$] was undertaken following the same adapted procedure.[1] Separately, $FeCl_3$ (2.70 g, 10 mmol) was dissolved in 50 mL of methanol and the NaDTC (6.76 g, 30

mmol) was dissolved in 150 mL of methanol, both with stirring. The solution of $FeCl_3$ was gradually added to the solution of NaDTC while under constant stirring. This was left for 2 hours, and the resultant precipitate collected under vacuum filtration. The black filtrate was dried under vacuum overnight and the resultant dry powder used in subsequent investigations. No further purification step was required. Yield 88%. Elemental analysis found (expected for $Fe(C_{15}H_{30}N_3S_6)$. C: 35.3 (35.9), H: 6.0 (6.0), N: 8.2 (8.4), S: 37.8 (38.3). IR (cm^{-1}): 462, 572, 599, 783, 845, 912, 994, 1073, 1094, 1133, 1145, 1207, 1268, 1294, 1354, 1373, 1432, 1449, 1486, 2867, 2929, 2973. m/z: 500.0.

Synthesis of Cu(diethyldithiocarbamate)$_2$ ([Cu(DTC)$_2$])

Synthesis of [Cu(DTC)$_3$] was undertaken following the same adapted procedure.[1] Separately, $CuCl_2$ (1.34 g, 10 mmol) was dissolved in 50 mL of methanol and the NaDTC (4.50 g, 20 mmol) was dissolved in 100 mL of methanol, both with stirring. The solution of $CuCl_2$ was gradually added to the solution of NaDTC while under constant stirring. This was left for 2 hours, and the resultant precipitate collected under vacuum filtration. The black filtrate was dried under vacuum overnight and the resultant dry powder used in subsequent investigations. Yield 94%. Elemental analysis found (expected for $Cu(C_{10}H_{20}N_2S_4)$. C: 33.4 (33.3), H: 5.6 (5.6), N: 7.8 (7.8), S: 35.7 (35.5). IR (cm^{-1}): 498, 571, 778, 846, 912, 995, 1072, 1096, 1146, 1206, 1271, 1300, 1352, 1376, 1434, 1451, 1592, 2867, 2928, 2973. m/z: 360.0.

Analysis of Silver(diethyldithiocarbamate) ([Ag(DTC)]

Purchased [Ag(DTC)] was characterised by elemental analysis found (expected for $Ag(C_5H_{10}N_1S_2) \cdot H_2O$; C: 21.8 (21.9), H: 3.8 (3.6), N: 5.4 (5.1), S: 23.3 (23.3). IR (cm^{-1}): 428,

504, 556, 776, 835, 899, 971, 1062, 1073, 1092, 1137, 1197, 1263, 1294, 1350, 1375, 1421, 1450, 1487, 2868, 2925, 2968.

Synthesis of Zn(diethyldithiocarbamate)$_2$ ([Zn(DTC)$_2$])

Synthesis of [Zn(DTC)$_2$] was undertaken following the same adapted procedure.[1] Separately, $ZnCl_2$ (1.36 g, 10 mmol) was dissolved in 50 mL of methanol and the NaDTC (4.50 g, 20 mmol) was dissolved in 100 mL of methanol, both with stirring. The solution of $ZnCl_2$ was gradually added to the solution of NaDTC while under constant stirring. This was left for 2 hours, and the resultant precipitate collected under vacuum filtration. The white filtrate was dried under vacuum overnight and the resultant dry powder used in subsequent investigations. Yield 90%. Elemental analysis found (expected for $Zn(C_{10}H_{20}N_2S_4)$. C: 33.3 (33.1), H: 5.6 (5.5), N: 7.7 (7.7), S: 35.6 (35.4). IR (cm^{-1}): 426, 505, 563, 777, 839, 910, 991, 1071, 1095, 1144, 1200, 1270, 1297, 1353, 1376, 1427, 1449, 1499, 2869, 2929, 2967. ^{1}H NMR (400 MHz, DMSO): δ 3.80 (q, 4 H), 1.20 (t, 6 H). m/z: 363.0.

Synthesis of Cd(diethyldithiocarbamate)$_2$ ([Cd(DTC)$_2$])

Synthesis of [Cd(DTC)$_2$] was undertaken following the same adapted procedure.[1] Separately, $Cd(NO_3)_2$ (3.08 g, 10 mmol) was dissolved in 50 mL of methanol and the NaDTC (4.50 g, 20 mmol) was dissolved in 100 mL of methanol, both with stirring. The solution of $Cd(NO_3)_2$ was gradually added to the solution of NaDTC while under constant stirring. This was left for 2 hours, and the resultant precipitate collected under vacuum filtration. The white filtrate was dried under vacuum overnight and the resultant dry powder used in subsequent investigations. Yield 91%. Elemental analysis found (expected for $Cd(C_{10}H_{20}N_2S_4)$. H: 4.9 (4.9), N: 6.8 (6.8), S: 31.3 (31.3), Cd: 27.5 (27.5). IR (cm^{-1}): 427, 505, 561, 776, 837, 910, 985, 1071, 1094, 1144,

1199, 1267, 1297, 1350, 1376, 1422, 1433, 1456, 1495, 2868, 2927, 2963, 2978. ^{1}H NMR (400 MHz, DMSO): δ 3.83 (q, 4 H), 1.21 (t, 6 H).

Synthesis of metal sulfides

Three metal sulfides were targeted in this study: (MnFeCuAgZnCd)S, (MnFeZnCd)S and Ag_3CuS_2. Synthesis of the these were undertaken using a previously reported literature procedure.[2] Each metal dithiocarbamate precursor (see SI for full synthetic details) was dissolved in DCM in a 1 : 1 : 1 : 1 : 1 : 1 molar ratio for the (MnFeCuAgZnCd)S, 1 : 1 : 1 : 1 molar ratio for the (MnFeZnCd)S and 3 : 1 molar ratio for the Ag_3CuS_2. The solvent was evaporated in a ceramic boat to leave a powder containing a homogenously dispersed mixture of all precursors. The ceramic boat containing the mixed powder was then placed in a tube furnace and under an Ar atmosphere and thermally treated at 500 °C for 1 h, with a ramp rate of 15 °C min^{-1}. The resultant powder was then collected and analysed.

High-pressure testing of the metal sulfides

Two types of experiments were performed as part of this study: high-pressure resistance measurements and pressure annealing experiments. Both types of experiments were performed in the same apparatus with very similar sample assemblies.

The three samples were compressed using a Walker type multi-anvil apparatus.[3,4] These apparatus compresses an octahedral sample assembly between 8 tungsten carbide cubes, each of which has a corner truncation. These cubes are in turn acted on by 6 outer-, 1st stage wedges. A 1000-tonne force load frame applies force to the 6 outer-wedges. The apparatus is arranged so that the force is focused through the different stages onto the sample assemblage in the middle.

The sample assemblage was an 18 mm, Cr-copped MgO octahedron with a 6.5 mm outer dimension, 4 mm inner dimension ZrO_2 sleeve inserted through a hold between opposing faces. For the resistance experiments, two MgO cylinders with inconel electrodes attached to

one side of them were inserted to fill the space. The HE powder was packed into an *ca.* 1 mm wide, 0.5 mm deep trench cut into the face of one of the MgO cylinders and was electrically connected to the outside by the Inconel electrodes.

Samples for the pressure annealing experiments were made by packing HE powder materials into gold-foil capsules. The capsules were *ca.* 2 mm diameter and *ca.* 3 mm long with a wall thickness of 50 μm (2× wraps of 25 μm gold).

Figure of cell assemblages.

Each experiment was compressed to the target pressure over a period of 2 – 3 h. During resistance experiments the resistance across the sample assemblage was recorded manually using readings from a high precision digital multimeter. Upon arrival at the target pressure, the pressure annealing experiments were left for 100 – 120 h at pressure. The pressure was removed from all the experiments over 2 – 3 h and the resistance recorded as part of the experiment. The recovered pressure anneal samples were recovered and prepared for pXRD, Raman, and TEM analysis. The recovered samples were hard enough for the gold foil capsule to be unrolled from the sample leaving a hard pellet. Half of each pellet was ground into a fine powder for pXRD and Raman spectroscopy and the remaining half was sectioned for TEM analysis using an ultramicrotome, resulting in ~50 nm sections.

Transmission electron microscopy and sample preparation

TEM samples were prepared by ultramicrotomy. For the high-pressure samples, the compressed pellet was embedded in mounting resin and a diamond knife used to cut slices with approximate thickness of 200, 100 and 50 nm, of these, the 50 nm slice was found to be the most useful for (S)TEM analysis. For the uncompressed powder sample the as-prepared

powder was mixed into the mounting resin and the resulting composite was sectioned by ultramicrotomy in the same way.

FIB sectioning was not considered as there were concerns about damage or recrystallisation of the samples from either the ion-beam energy deposition or from gallium ion implantation.

STEM-EDX clustering

The STEM EDX datasets were clustered using a 2-stage process based on that developed for similar data analysis.[10] Raw STEM-EDX spectra were truncated to 10 KeV (2000 energy channels) and were subsequently normalised using the RobustScalar procedure[11] (subtracting the median value and dividing by the inter-quartile range). The scaled data was then analysed using principle-component analysis (PCA) and the 'scree' plot used to identify the number of latent variables needed to describe most of the structured variance in the data. Typically, this was found to be 6 components and this standard value was used for all scans.

The loading values for the PCA model were then used to construct a low-dimensional latent space within which a Gaussian mixture model (GMM)[11] was applied to identify clusters of similar spectra. A range of cluster numbers between 2 and 10 were attempted for all the experimental STEM -EDX scans and the final number of clusters refined manually by comparing both the spatial localisation as well as the cluster average spectra to tell where sub-clustering was occurring.

The resulting cluster average spectra were then quantified using a Cliff-Lorimer K-factor analysis implemented in the Hyperspy spectral analysis package.[12] 200kV $K_{A,Si}$ values were used for the analysis taken from published sources.[13]

Characterisation of (MnFeCuAgZnCd)S prior to pressure annealing

While it is impossible to accurately determine grain size, since there appears to have been some fracturing and shearing of material during sectioning, it seems reasonable to estimate a primary grain size between several hundred nm up to a few μm in (MnFeCuAgZnCd)S prior to pressure annealing. The post-pressure annealed (MnFeCuAgZnCd)S material was found to have grain sizes significantly smaller, on the order of 10 – 100 nm.

Powder X-ray diffraction

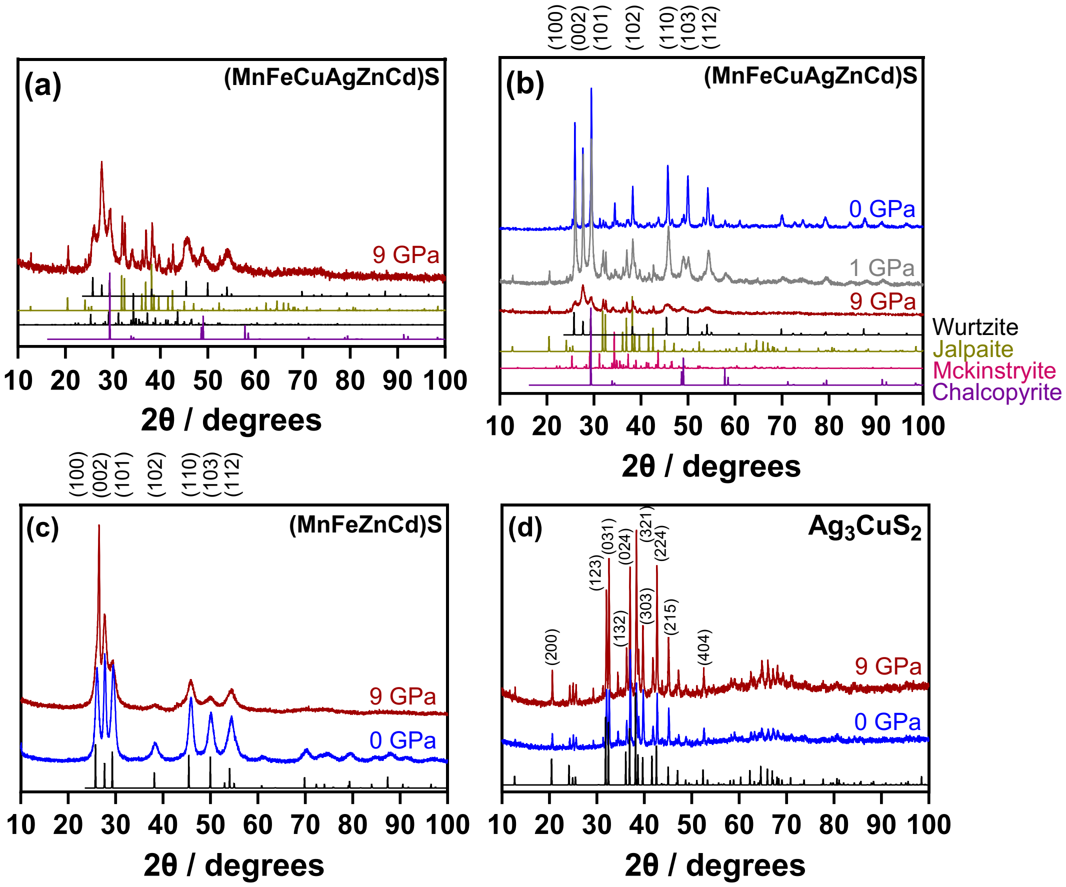

Figure S1. pXRD patterns of (a, b) (MnFeCuAgZnCd)S, (c) (MnFeZnCd)S and (d) Ag_3CuS_2 both before (blue) and after (red) subjection to 9 GPa of pressure-annealing. Also shown in (a, b) in grey is the pXRD pattern when (MnFeCuAgZnCd)S is subjected to 1 GPa of pressure-annealing. Model patterns correspond to wurtzite (ICSD: 67453) (in (a, b, c)), jalpaite (ICSD: 67526) (in (a, b, d) and chalcopyrite (ICSD: 2516) (in (a, b)) with miller indices indicated.

Raman spectroscopy

Raman spectroscopy found that for the (MnFeCuAgZnCd)S system (Figure S2), two peaks centred at *ca.* 300 cm^{-1} and *ca.* 600 cm^{-1} were present, which are consistent with the first and second longitudinal optical (LO) modes of hexagonal wurtzite CdS,[5,6] again supporting our classification of the major phase present as Wurtzite. The Raman spectra of Ag_3CuS_2 found two peaks centred at *ca.* 260 cm^{-1} and 470 cm^{-1}.

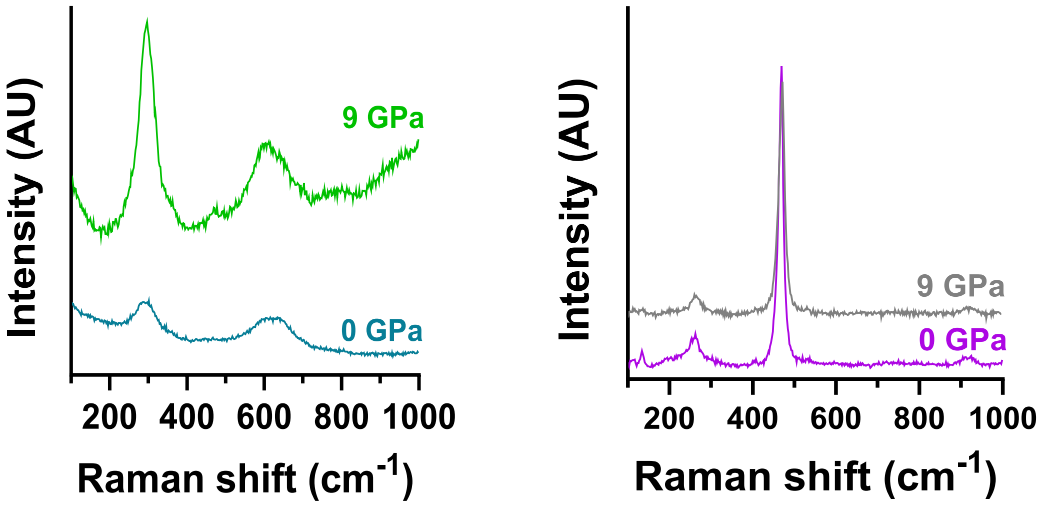

Figure S2. Raman Spectra of (left) (MnFeCuAgZnCd)S and (right) Ag_3CuS_2 both before pressure (0 GPa) and after high pressure (9 GPa).

Scanning electron microscopy – energy dispersive X-ray spectroscopy (SEM-EDX)

SEM-EDX was used to assess the microscale elemental homogenisation within the three materials. For (MnFeCuAgZnCd)S (Figure S3), some localisation of Fe and Cu (consistent with chalcopyrite) was potentially present. However, there wasn't any notable localisation of Ag and Cu, despite peaks in the pXRD pattern indicating Jalpaite (Ag_3CuS_2) formation. For both (MnFeZnCd)S (Figure S4) and Ag_3CuS_2 (Figure S5), all elements appeared to be homogenously distributed at the microscale.

(MnFeCuAgZnCd)S

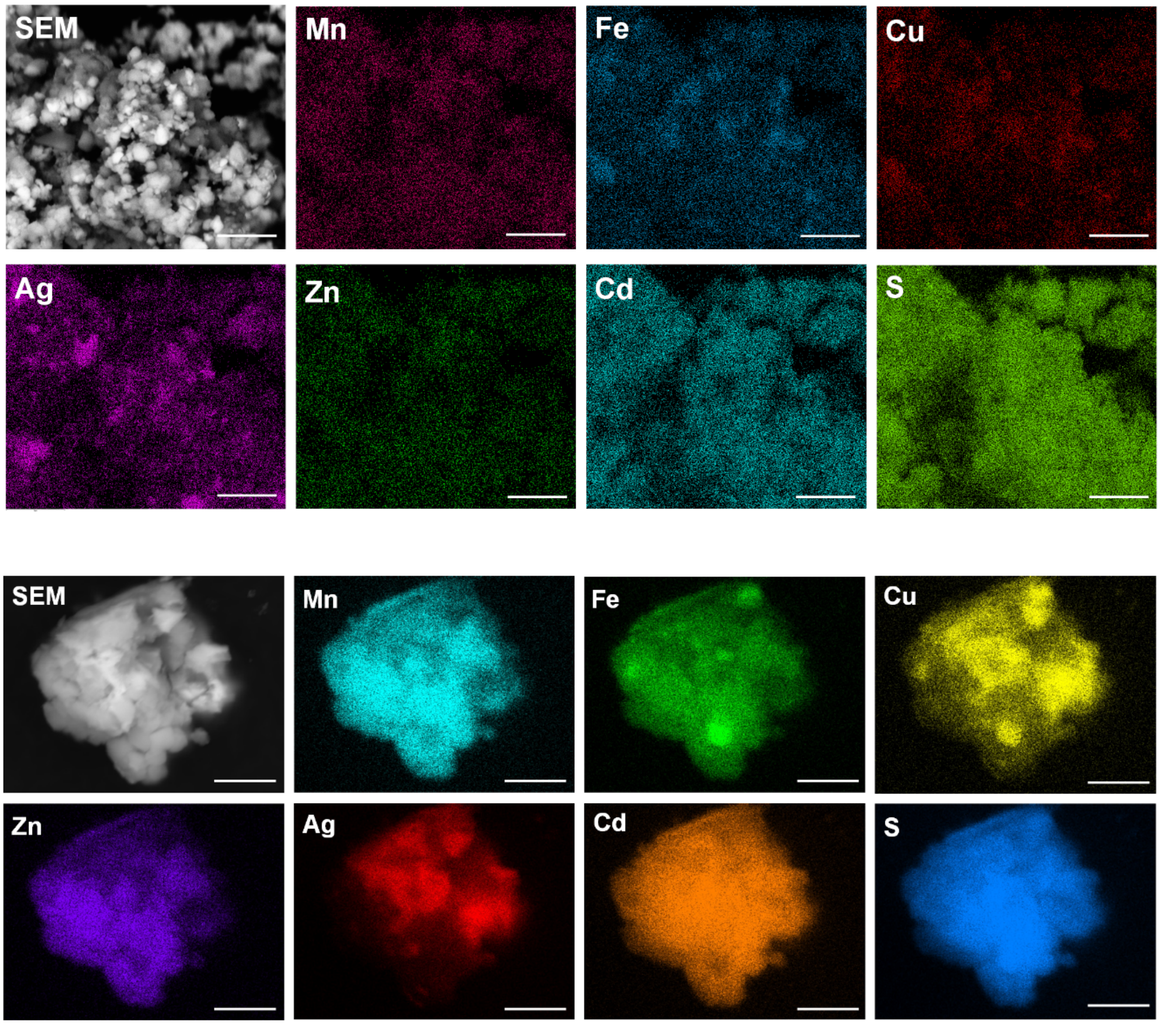

Figure S3. SEM-EDX map of (MnFeCuAgZnCd)S recorded at 20 eV (above) before and (below) after being subjected to 9 GPa of pressure-annealing, showing a scale bar of 5 μm.

Table S1. Table of data showing SEM-EDX elemental composition analysis of (MnFeCuAgZnCd)S before and after pressure-annealing.

Element	Expected / %	Before Pressure / At.%	After Pressure / At.%
**Mn**	8.3	9	9
**Fe**	8.3	10	8
**Cu**	8.3	9	8
**Ag**	8.3	10	11
**Zn**	8.3	8	8
**Cd**	8.3	10	10
**S**	50	44	45

(MnFeZnCd)S

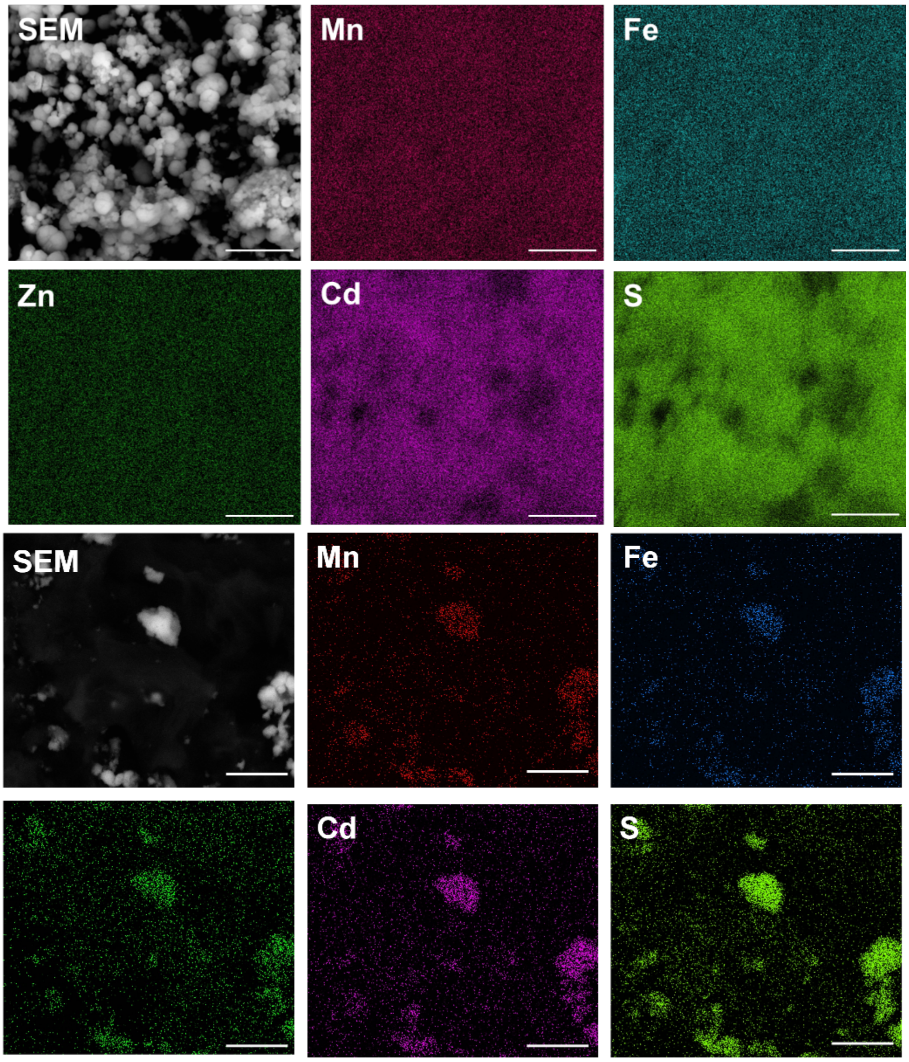

Figure S4. SEM-EDX map of (MnFeZnCd)S recorded at 20 eV (above) before and (below) after being subjected to 9 GPa of pressure-annealing, showing a scale bar of 5 μm.

Table S2. Table of data showing SEM-EDX elemental composition analysis of (MnFeZnCd)S before and after pressure-annealing.

Element	Expected / %	Before Pressure / At.%	After Pressure / At.%
**Mn**	13	14	13
**Fe**	13	13	10
**Zn**	13	11	11
**Cd**	13	14	13
**S**	50	48	53

Ag_3CuS_2

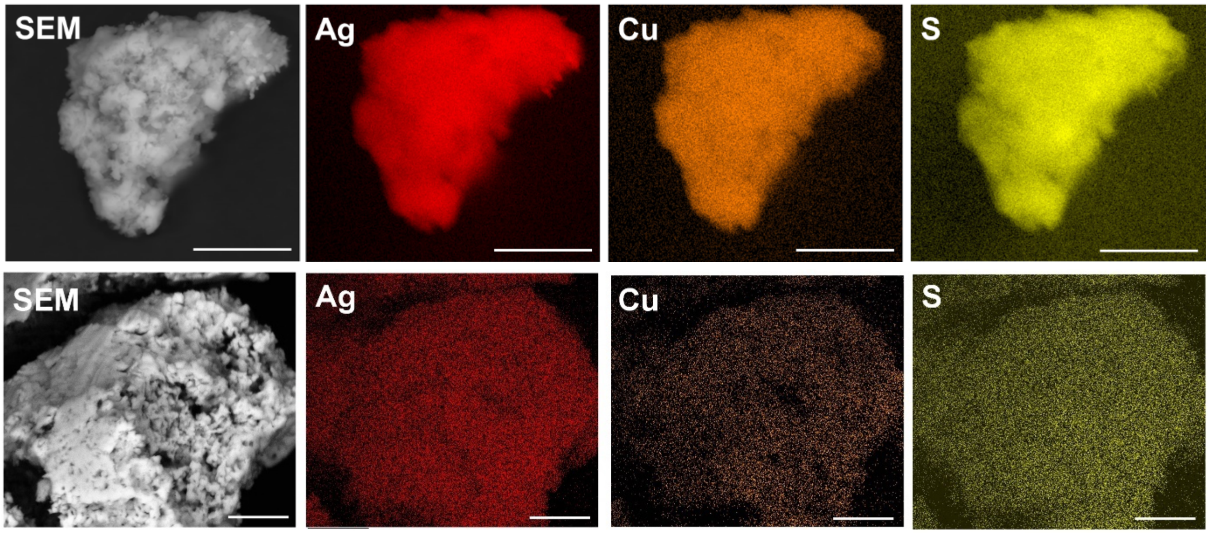

Figure S5. SEM-EDX map of Ag_3CuS_2 recorded at 20 eV (above) before and (below) after being subjected to 9 GPa of pressure-annealing, showing a scale bar of 5 μm.

Table S3. Table of data showing SEM-EDX elemental composition analysis of Ag_3CuS_2 before and after pressure-annealing.

Element	Expected / %	Before Pressure / At.%	After Pressure / At.%
**Ag**	50	47	48
**Cu**	17	17	19
**S**	33	36	33

Higher pressure on Ag_3CuS_2

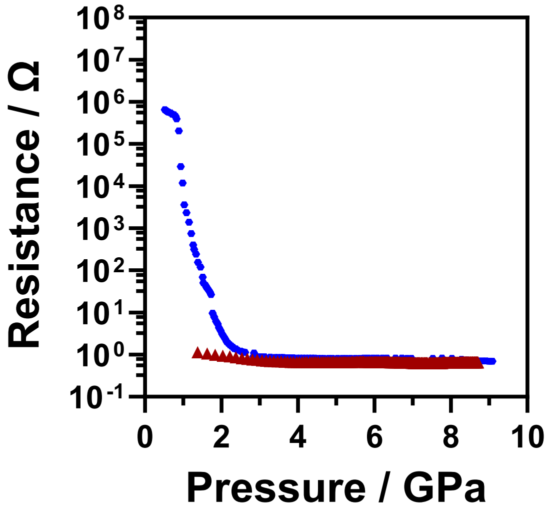

Figure S6. Figure showing the resistance with increasing pressure of Ag_3CuS_2 whereby there is no significant change in resistance above 3 GPa (Figure 2(c) in the main text).

Characterisation of (MnFeCuAgZnCd)S prior to pressure annealing

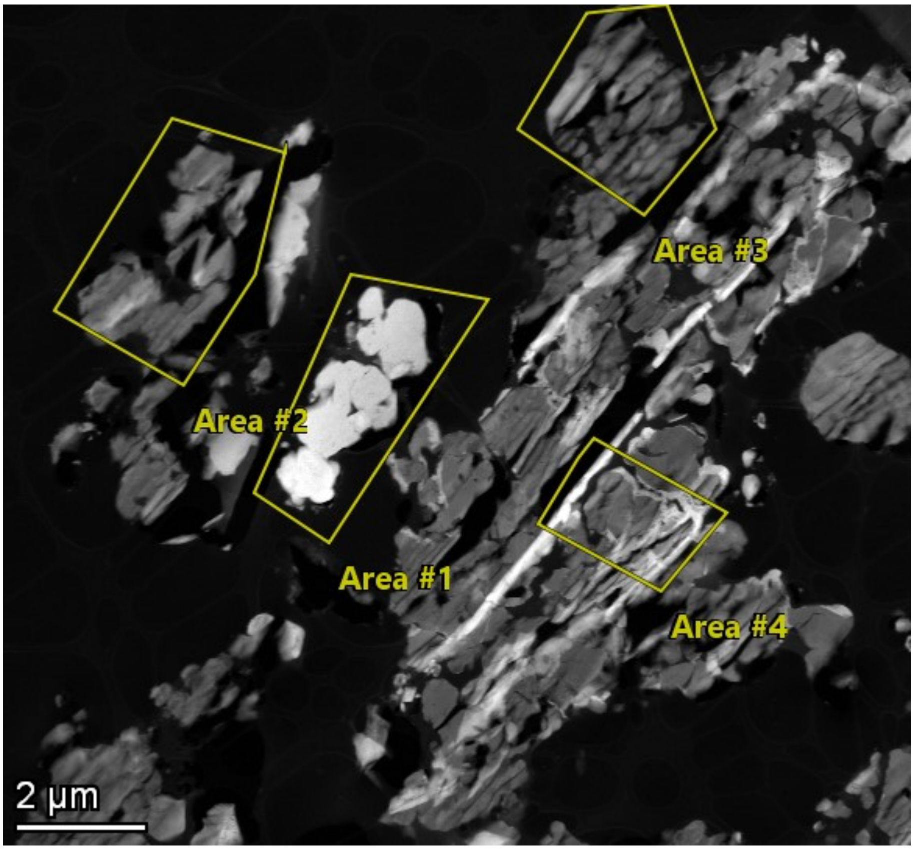

Figure S7. HAADF-STEM image showing four regions analysed in Figure 2 and quantified in Table 1 in the main text.

Further STEM-EDX clustering

Multiple regions of the post-pressure annealed (MnFeCuAgZnCd)S material was analysed to confirm the utility of this analysis method.

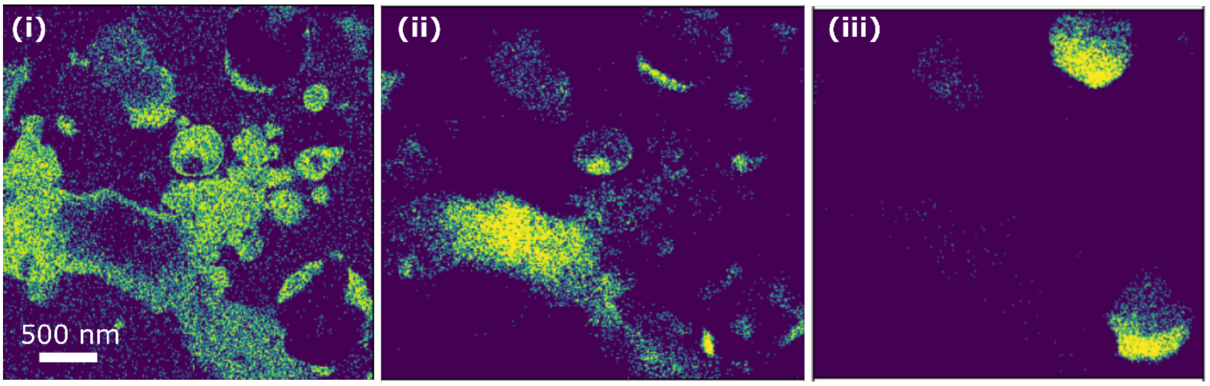

Figure S8. STEM-EDX clustering outputs for a region of the post-pressure sample. Comprising 3 major phases: (i) wurtzite matrix, (ii) exsolved jalpaite and (iii) retained chalcopyrite. Composition analysis given in Table S4.

Table S4. Quantification of the STEM-EDX clustering in Figure S8.

	**Cluster i (wurtzite)**	**Cluster ii (jalpaite)**	**Cluster iii (chalcopyrite)**
**Ag**	16.7 ± 1.2	27.1 ± 1.6	2.1 ± 0.3
**Cd**	16.8 ± 1.7	20.4 ± 1.4	1.5 ± 0.2
**Cu**	30.0 ± 1.9	33.7 ± 2.1	46.1 ± 2.3
**Fe**	10.1 ± 0.8	5.1 ± 0.5	43.1 ± 2.2
**Mn**	11.7 ± 0.9	6.7 ± 0.6	6.5 ± 0.7
**Zn**	14.7 ± 0.2	7.1 ± 0.1	0.5 ± 0.5

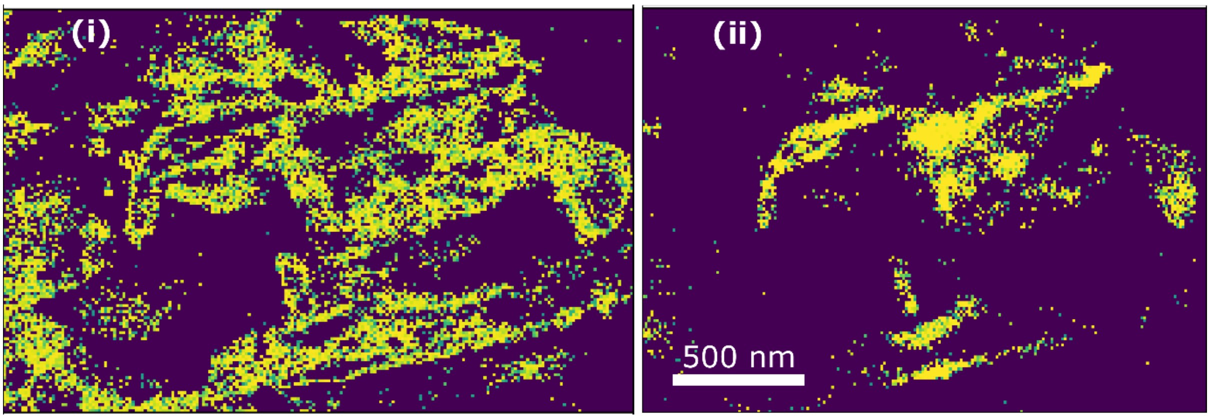

Figure S9. STEM-EDX clustering outputs for a region of the post-pressure sample. Comprising 2 major phases: (i) wurtzite matrix and (ii) exsolved jalpaite. Composition analysis given in Table S5.

Table S5. Quantification of the STEM-EDX clustering in Figure S9.

	**Cluster i (wurtzite)**	**Cluster ii (jalpaite)**
**Ag**	3.6 ± 0.2	13.4 ± 1.1
**Cd**	16.5 ± 1.7	16.8 ± 1.1
**Cu**	27.8 ± 1.9	26.1 ± 1.7
**Fe**	15.4 ± 1.3	15.9 ± 1.2
**Mn**	19.5 ± 1.2	14.7 ± 1.1
**Zn**	17.1 ± 1.2	13.0 ± 1.1

Analysis of (MnFeZnCd)S

Pre-pressure annealing

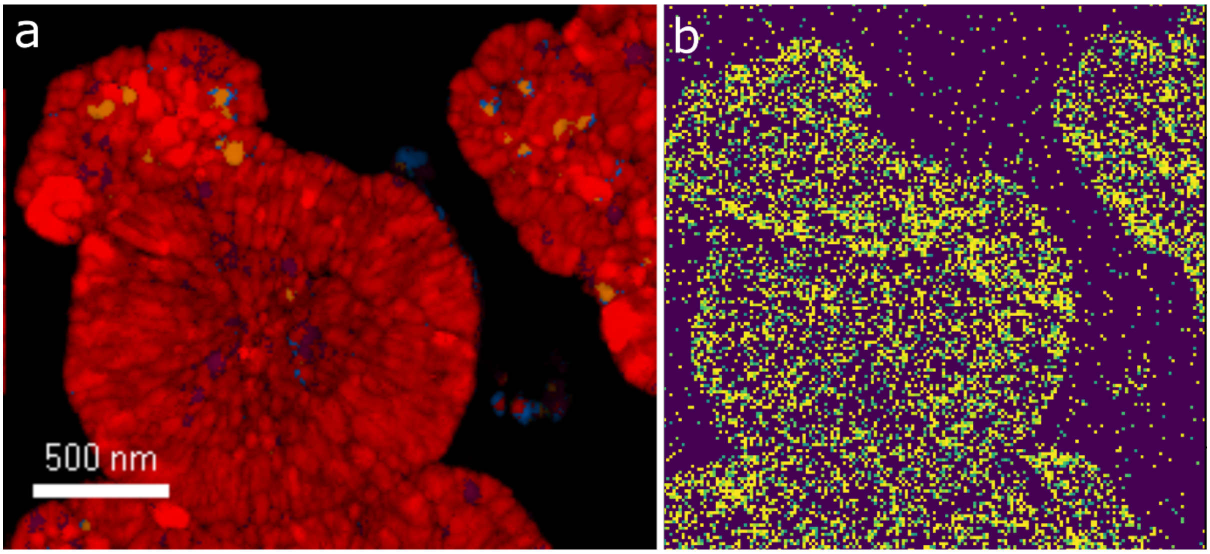

Figure S10. (a) Phase mapping of the pristine 4-element system, red indicates wurtzite, purple indicates pyrrhotite and orange indicates pyrite. (b) shows a single STEM-EDX cluster for the entire region. Quantification for the cluster is shown in Table S6 below.

Table S6. Quantification of the STEM-EDX clustering in Figure S10.

	**Cluster (wurtzite)**
**Cd**	23.3 ± 1.2
**Fe**	28.1 ± 0.9
**Mn**	25.7 ± 0.7
**Zn**	22.8 ± 0.6

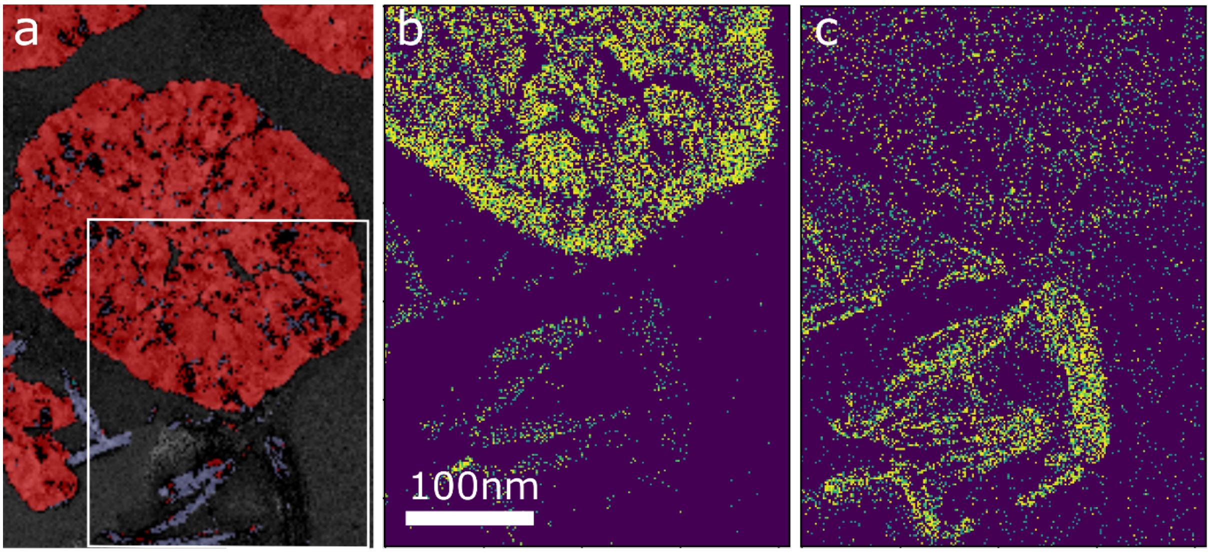

Figure S11. (a) Phase mapping of the pristine 4-element system, red indicates wurtzite, purple indicates pyrrhotite and orange indicates pyrite. The outlined region is the approximate area used for STEM-EDX experiments. (b and c) show STEM-EDX clustering outputs corresponding to the majority wurtzite phase and a trace impurity of pyrrhotite respectively. Quantification for the cluster is shown in Table S7.

Table S7. Quantification of the STEM-EDX clustering in Figure S11.

	Cluster (b) (wurtzite)	Cluster (c) (pyrrhotite)
**Cd**	20.1 ± 0.4	0.9 ± 0.1
**Fe**	32.9 ± 0.8	95.7 ± 3.5
**Mn**	25.5 ± 0.8	2.8 ± 0.3
**Zn**	22.8 ± 0.6	0.6 ± 0.1

The majority of the pristine sample is attributable to the wurtzite phase with broadly even distribution of the 4 cations. The secondary pyrrhotite phase was only found in this one location of the TEM grid analysed, suggesting it is a small-scale impurity in the system, which explains why it was not detected in the initial pXRD analysis.

Post-pressurised 4-element samples

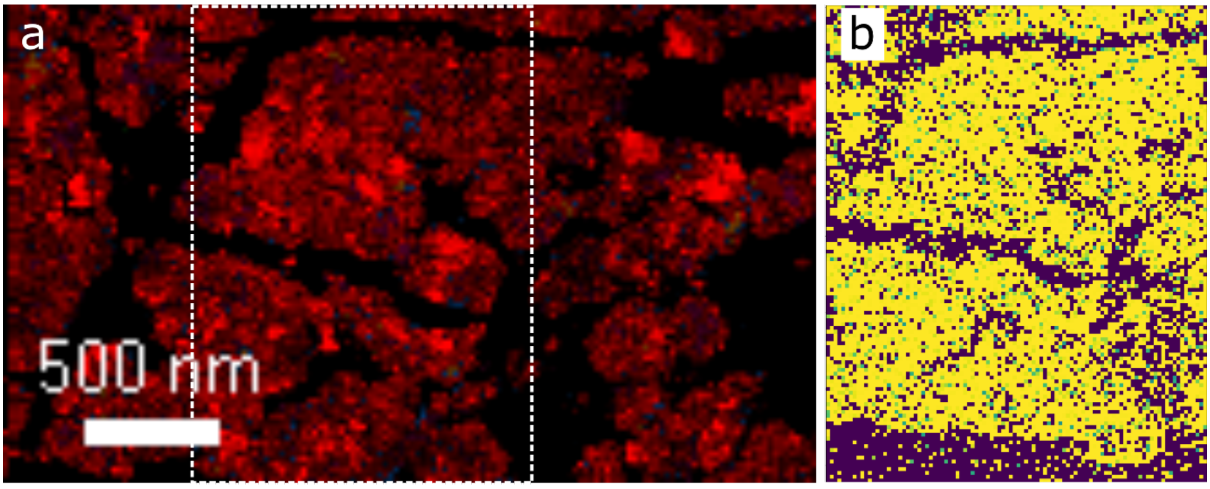

Figure S12. (a) Phase mapping of the pressurised 4-element system, red indicates wurtzite, purple indicates pyrrhotite and orange indicates pyrite. The outlined region is the approximate area used for STEM-EDX experiments. (b) shows STEM-EDX clustering outputs indicating an overwhelming single phase in the sample. Quantification for the cluster is shown in Table S8.

Table S8. Quantification of the STEM-EDX clustering in Figure S12.

	**Cluster (b) (wurtzite)**
**Cd**	20.7 ± 0.4
**Fe**	30.9 ± 0.6
**Mn**	25.6 ± 0.4
**Zn**	22.7 ± 0.3

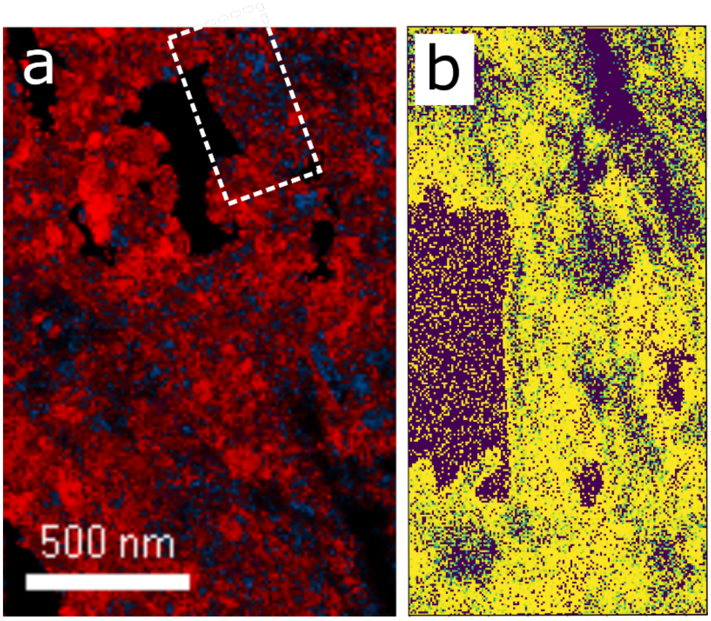

Figure S13. (a) Phase mapping of the pressurised 4-element system, red indicates wurtzite, purple indicates pyrrhotite and orange indicates pyrite. The outlined region is the approximate area used for STEM-EDX experiments. (b) shows STEM-EDX clustering outputs corresponding to a wurtzite phase. Quantification for the cluster is shown in Table S9.

Table S9. Quantification of the STEM-EDX clustering in Figure S13.

	**Cluster b (wurtzite)**
**Cd**	24.4 ± 0.6
**Fe**	30.4 ± 0.5
**Mn**	22.2 ± 0.3
**Zn**	23.0 ± 0.3

The post pressure samples show a nearly identical majority wurtzite phase distribution. There is potential evidence for pyrrhotite inclusions in Figure S10, but in most cases these positions returned a poor cross-correlation score during template matching and in the EDX data (Figure S10(b)) they correspond with holes in the film suggesting the diffraction indexing may be erroneous. The post-pressure samples have a general cation stoichiometry consistent with the pristine material on the 10s of nm scale, suggesting there is no reordering of the cations in the

material at this length scale, but we cannot exclude this being the case at the unit cell (~Å) level. The only change is that instead of broadly micron-sized spherical agglomerates of wurtzite crystals in the pristine material, the pressurised sample was a compacted pellet. There is no clear evidence of a change in the average crystallite size of 40 – 50 nm in each situation, though the pressurised sample appears to have fractured more unevenly during sectioning compared to the pristine material.

Scanning precessional electron diffraction (SPED) patterns

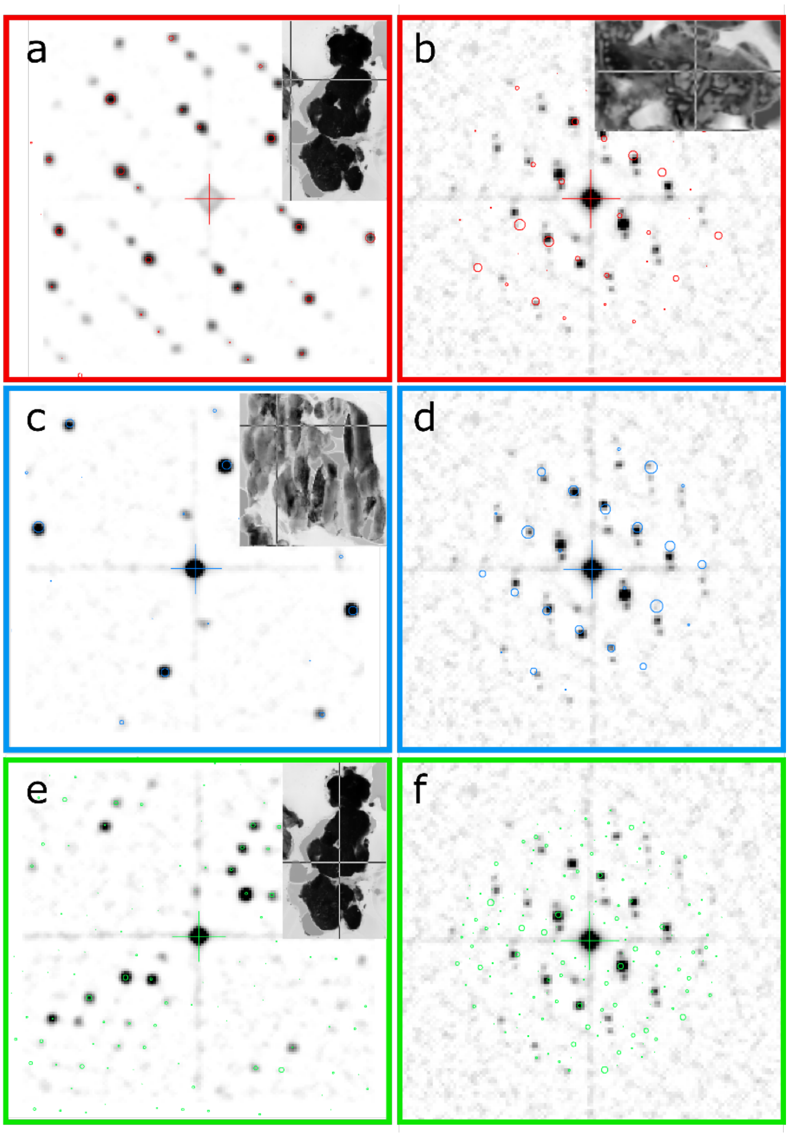

Figure S14. Scanning precessional electron diffraction (SPED) patterns of (MnFeCuAgZnCd)S, overlaid with template matches. a) and b) show patterns from the pre- and post-annealed sample respectively (the position of the pattern in the SPED scan are shown inset. c) and d) show similar pattern matches for chalcopyrite and e) and f) are for jalpaite.

SPED phase analysis results are shown in Figure S14. The left-hand column (a, c and e) show patterns from the pre-annealed sample. In all cases a unique phase and orientation solution was possible. For the wurtzite region the crystal orientation was found to be close to <631>, for chalcopyrite the orientation was close to <951> and for jalpaite the orientation was <968> (this is only a few degrees misoriented from <111>). For the post-annealing data, the same pattern was compared to all three libraries of patterns. The best-fit was found for wurtzite (Figure S14(b)) with an orientation of <312>, but matches with similar cross correlation scores were also found for chalcopyrite <203> (Figure S14(d)) and jalpaite <814> (Figure S14(f)).

References

1 F. Makin, F. Alam, M. A. Buckingham and D. J. Lewis, *Scientific Reports*, 2022, **12**, 5627.

2 W. Xiao, Y. Li, A. Elgendy, E. C. Duran, M. A. Buckingham, B. F. Spencer, B. Han, F. Alam, X. Zhong, S. H. Cartmell, R. J. Cernik, A. S. Eggeman, R. A. W. Dryfe and D. J. Lewis, *Chem. Mater.*, 2023, **35**, 7904–7914.

3 D. Walker, M. A. Carpenter and C. M. Hitch, *American Mineralogist*, 1990, **75**, 1020–1028.

4 H. Keppler and D. J. Frost, *Mineral Behaviour at Extreme Conditions*, European Minerologist Union, 2005, vol. 7.

5 M. A. Buckingham, A. L. Catherall, M. S. Hill, A. L. Johnson and J. D. Parish, *Crystal Growth and Design*, 2017, **17**, 907.

6 M. A. Buckingham, K. Norton, P. D. McNaughter, G. Whitehead, I. Vitorica-Yrezabal, F. Alam, K. Laws and D. J. Lewis, *Inorg. Chem.*, 2022, **61**, 8206–8216.

7 E H. Kisi and M. M. Elcombe. *Acta Crystallographica*, 1989, C**45**, 1867-1870

8 K. S. Knight, W. G. Marshall and S. W. Zochowski. *The Canadian Mineralogist*, 2011, **49**, 1015-1034.

9 C. L. Baker, F. J. Lincoln and W. S. Johnson W. S. Australian Journal of Chemistry. 1992, 45, 1441-1449.

10 E. C. Duran, Z. Kho, J. F. Einsle, I. Azaceta, S. A. Cavill, A. Kerrigan, V. K. Lazarov and A. S. Eggeman. *Computational Materials Science*, 2023, **228**, 112336

11 F. Pedregosa, G. Varoquaux, A. Gramfort, V. Michel, B. Thirion, O. Grisel and M Blondel. *Journal of Machine Learning Research*, 2011, **12**, 2825-2830

12 https://doi.org/10.5281/zenodo.11148112

13 P. J. Sheridan. *J. Electr. Microsc. Tech.* 1989, **11**, 41–61.
